# Designing multifunctional recombinant vaccines: an engineering strategy based on innovative epitope prediction-guided splicing

**DOI:** 10.7150/thno.103755

**Published:** 2025-03-03

**Authors:** Zhidong Wang, Xiaolin Yang, Xiaoyi Wei, Licai Shi, Xuechun Wang, Zhenjian Zhuo, Hailong Su, Wengao Wu, Yu J. Cao

**Affiliations:** 1 State Key Laboratory of Chemical Oncogenomics, Shenzhen Key Laboratory of Chemical Genomics, Peking University Shenzhen Graduate School, Shenzhen, Guangdong, 518055, China.; 2 School of Laboratory Medicine and Biotechnology, Southern Medical University, Guangzhou, China.; 3 Department of Cardiovascular Surgery, Yueyang Central Hospital, Yueyang, Hunan, 414000, China.; 4 Institute of Chemical Biology, Shenzhen Bay Laboratory, Shenzhen, 518132, China.

**Keywords:** recombinant vaccine, epitope prediction, immunogenicity enhancing, nasopharyngeal carcinoma, immune checkpoint inhibitor combination

## Abstract

**Background:** Recombinant subunit vaccines leveraging pathogen-derived components are pivotal for disease prevention. Nonetheless, application of these vaccines still faces challenges such as low immunogenicity and a short half-life. Additionally, selecting appropriate antigens presents a significant barrier in recombinant vaccine design.

**Methods:** Here, we applied a novel approach to address these challenges by developing recombinant vaccines targeting LMP2A. We employed *in silico* epitope prediction and splicing to create epitope enrichment regions (EERs) in conjunction with the TLR4 agonist hEDA to enhance immunogenicity and the immunoglobulin G1 (IgG1) Fc fragment to prolong persistence.

**Results:** This multifaceted strategy enhances antigen uptake by antigen-presenting cells, eliciting major histocompatibility complex (MHC) allele-dependent T-cell responses against targeted epitopes. Compared with split-component candidates, these innovatively designed vaccines demonstrate superiority in inducing the development of IFN-γ^+^ antigen-specific T cells, along with elevated humoral and cellular immune responses, and exhibit significantly enhanced antitumor efficacy in both preventive and therapeutic models. Furthermore, optimized vaccine treatment synergistically inhibits tumor growth when combined with the administration of immune checkpoint inhibitors, leading to significantly prolonged survival.

**Conclusion:** This novel design strategy offers advances for the development of multifunctional recombinant vaccines and represents a promising platform for cancer immunotherapy and applications in other diseases.

## Introduction

Since the development of the smallpox vaccine by Edward Jenner in 1796, vaccines have played a pivotal role in safeguarding populations against infectious diseases and tumors 1. Recombinant subunit vaccines, comprising small components derived from pathogens, have emerged as a popular design for modern vaccines 2. Among these, the hepatitis B vaccine has an 84% coverage rate, indicating its widespread use in routine vaccination programs worldwide 3, 4. The development of these vaccines relies on the identification of pathogen components that elicit protective immunity post-infection 5, 6. Therefore, accurate identification of these components responsible for infection, progression and tumorigenesis allows for the simplification and optimization of vaccine formulation development 7, 8. Typically, antigens in recombinant subunit vaccines consist of whole antigen proteins, such as the SARS-CoV-2 spike protein, hepatitis B surface antigen, and Epstein‒Barr virus (EBV) gp350 or its extracellular domain 9-12. Given that additional domains beyond antigen epitopes have the potential to induce allergic reactions 13, 14, accurately identifying antigen epitopes and carefully fusing multiple epitopes will not only reduce the likelihood of allergic reactions but also enhance efficiency *in vivo*.

*In silico* prediction-assisted epitope mining has proven to be efficient and instructive 15, aiding in the precise prediction of antigen components and their recognition. Despite the inherent complexity of antigen recognition and presentation processes, data-driven artificial intelligence sequence-based methods for prediction and molecular modeling significantly enhance predictive accuracy 16. Epitope prediction-assisted vaccine design has been successfully applied in the development of vaccines for SARS-CoV-2 and *Brucella melitensis* 17, 18. However, in complex physiological environments, single-epitope immunization may trigger a limited response 19, 20. To improve the effectiveness and expand the scope of protection, researchers frequently opt to combine multiple epitopes within a single molecule, with or without linkers 21, 22. A notable example is the influenza vaccine multimeric-001 developed by BiondVax Pharmaceuticals Ltd., which combines nine conserved epitopes in triplicate into one molecule, and this vaccine provides protection against various influenza strains 23, 24. Despite the benefits of epitope prediction in targeting specific epitopes, the inherent disadvantages of epitope-based recombinant vaccines, such as low immunogenicity and short half-life, persist.

Immunogenicity enhancement is achieved by incorporating adjuvants into vaccines to activate pattern recognition receptors (PRRs), thereby improving the uptake and presentation of vaccine antigens by antigen-presenting cells 25. Toll-like receptors (TLRs) are the most vital PRRs expressed on innate immune cells, and fusion with TLR agonists, such as defensin or the extra domain A of fibronectin (EDA) 26-29, has been an effective approach for enhancing both innate and adaptive immunity. Additionally, to prolong the half-life of vaccines, integration with nanoparticles or long-lasting molecules such as ferritin, human serum albumin or IgG1 Fc fragment has been immensely successful in the development of bioengineering platforms 30-32. Notably, a relatively long half-life and a robust immune response were observed with Fc fusion in recombinant vaccines for SARS-CoV-2 and EBV 33, 34. To confer dual functional enhancement to vaccines, materials science and multicomponent formulations are employed in vaccine design. A typical approach involves encapsulating the antigen with biocompatible materials to extend the half-life while concurrently incorporating noncovalently bound molecules, such as monophosphoryl lipid A or cyclic GMP-AMP, to enhance immunogenicity 35, 36. However, unlinked multicomponent formulations reduce consistency and increase manufacturing complexity. Therefore, there is an urgent need for a platform enabling the covalent fusion of immune-enhancing elements with specific antigen epitopes into a molecule with a clearly defined structure.

Viral infections play a crucial role in the tumorigenesis of several cancers, and vaccines targeting viral antigens have shown promise in enhancing treatment outcomes. For example, vaccines against human papillomavirus and hepatitis B virus have demonstrated significant clinical benefits 37, 38. In regions like Southeast Asia and North Africa, nasopharyngeal cancer (NPC), a prevalent type of head and neck cancer, exhibits a notably high incidence, with EBV infection strongly linked to its development 39. Despite this association, an effective vaccine specifically targeting NPC remains unavailable. Although EBV remains latent within NPC cells, several viral proteins, such as Epstein-Barr nuclear antigen 1, latent membrane protein 1, and latent membrane protein-2A (LMP2A), continue to be expressed and represent potential targets for vaccine development 40. Among these, LMP2A is particularly compelling due to its high tumor specificity and sustained expression 41. However, due to its unique transmembrane structure, vaccines targeting LMP2A are typically developed using viral vectors or nucleic acid platforms, which have demonstrated limited efficacy 42, 43. This highlights the urgent need for novel, more effective vaccines targeting LMP2A.

In this study, we established a platform for designing multifunctional recombinant vaccines targeting the EBV antigen LMP2A. *In silico* prediction was utilized to identify and combine adjacent epitopes to create EERs, and the TLR4 agonist human fibronectin EDA (hEDA) was incorporated to enhance immunogenicity, as was the human IgG1 Fc to prolong persistence. These novel vaccines facilitated improved antigen uptake in antigen-presenting cells and elicited MHC allele-dependent T-cell activities in response to targeted EERs both *in vitro* and *in vivo*. Given the association of EBV infection with NPC 39, we initially investigated the effects of these recombinant vaccines in preventive and therapeutic tumor models and demonstrated overall enhanced humoral and cellular immune responses. Notably, synergistic antitumor effects were observed when the vaccines were combined with a PD-1 inhibitor. Together, our studies provided innovative insights for the development of recombinant vaccines, highlighting the effectiveness of a multifunctional vaccine platform in bolstering multipronged protective responses against tumors and virulent pathogens.

## Results

### Designing recombinant subunit vaccines targeting LMP2A

**Figure 1:**
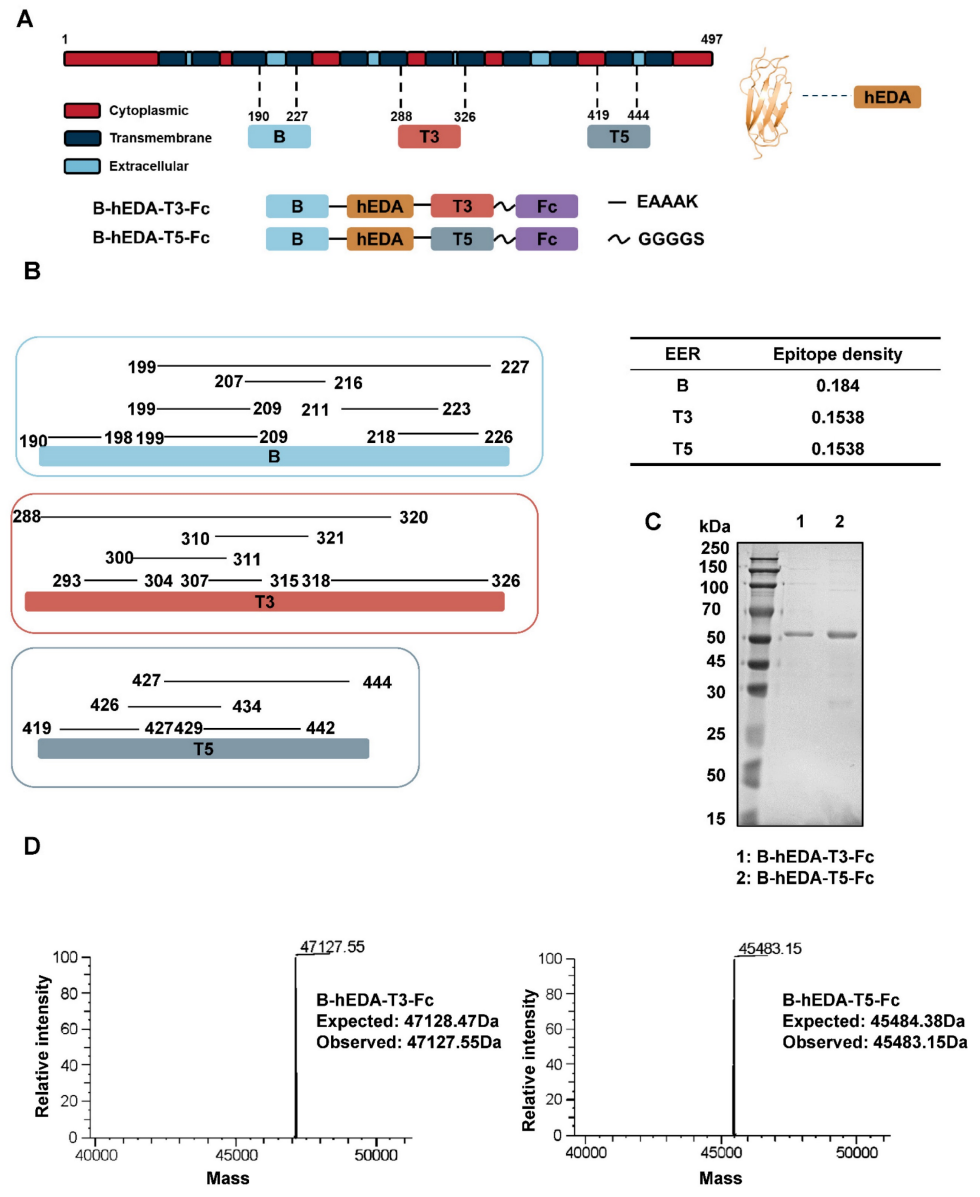
** Epitope prediction and design of LMP2A-targeted recombinant vaccines**. **A**) Schematic representation of the recombinant vaccines. The positions of B, T3 and T5 on the LMP2A sequence are indicated, along with the structural depiction of hEDA and the fusion approach for the two recombinant vaccines, B-hEDA-T3-Fc and B-hEDA-T5-Fc. **B**) Annotation of adjacent and overlapping epitopes in B, T3, and T5, along with detailed quantification of the epitope density of each EER. **C**) Evaluation of the purity and molecular weight of the recombinant vaccines using SDS-PAGE. The results of reducing SDS‒PAGE analyses with the molecular weight markers are shown. **D**) The molecular weights of the recombinant vaccines are accurately measured via LC-MS, and the results reveal that the observed molecular weights are consistent with the expected values.

Targeting NPC, we focused on the LMP2_EBVB9 (P13285) antigen and analyzed prevalent HLA alleles in Southeast Asia using the Allele Frequency Net Database. Prevalent HLA alleles, with a coverage rate exceeding 10% among Southeast Asian populations, were analyzed, encompassing all prevalent HLA-I and HLA-II subtypes (Figure S1). Using Immune Epitope Database (IEDB) toolkit, we predicted cytotoxic T lymphocyte (CTL) and helper T lymphocyte (HTL) epitopes with these MHC subtypes. We identified 79 epitopes after refining the CTL predictions with scores exceeding 0.5, and obtained 33 CTL epitopes after eliminating overlapping fragments (Table S1). Similarly, we derived 16 HTL epitopes from a predicted pool of 455 HTL epitopes by applying filters (IC50 < 50, PR < 10) (Table S2). To identify potential B-cell epitopes, we focused on two outer membrane loops of the twelve-pass transmembrane protein LMP2A: loop 2 (RIEDPPFNSLLFA, 199-211) and loop 5 (LQTNFKSLSSTEF, 376-388). Loop 5 was excluded for two reasons: first, it was not identified as a linear B-cell epitope, and second, it was predicted to have allergenic properties (Table S3). As a result, we selected Loop 2 (LMP2A 199-209) as the exclusive B-cell epitope, consistent with the findings reported by Zhang *et al.* 44. We combined adjacent epitopes with overlapping sequences to ensure that the epitope density per amino acid length exceeded 0.1, resulting in the formation of six EERs: EER 1 (residues 119-163), EER 2 (residues 254-291), EER 3 (residues 288-326), EER 4 (residues 345-392), EER 5 (residues 419-444) and EER 6 (residues 190-227) (Table S4). For instance, EER3 comprised six overlapping epitopes—residues 293-304, 300-311, 307-315, 310-321, 318-326 and 288-320—with an epitope density per amino acid length of 6/39 = 0.1538. To integrate humoral and cellular immunity, we fused EER 6 (representative of a B-cell epitope-based EER, designated B) with the other five T-cell epitope-based EERs (designated T1-5) in the presence of the TLR4 agonist hEDA, resulting in five LMP2A EER splicing candidates. Based on the structural differences predicted by Protein Homology/analog Y Recognition Engine V 2.0 (Figure S2), we selected two representative candidates, B-hEDA-T3 and B-hEDA-T5, and fused them with the human IgG1 Fc portion to generate two multifunctional recombinant vaccines, B-hEDA-T3-Fc and B-hEDA-T5-Fc. The locations of the B, T3 and T5 epitopes in LMP2A selected for vaccine design, along with the annotated overlapping sequences and epitope density, are illustrated in Figure 1A-B, respectively. For ease of detection, we added a Flag tag at the N-terminus of these two recombinant vaccine constructs. Following expression and purification, the vaccine antigens were analyzed via SDS-PAGE, which revealed an overall purity of 95% (Figure 1C), and LC-MS was used to confirm the expected molecular weight (Figure 1D).

### HLA isotype-dependent cellular immunity-inducing function of recombinant vaccines

**Figure 2:**
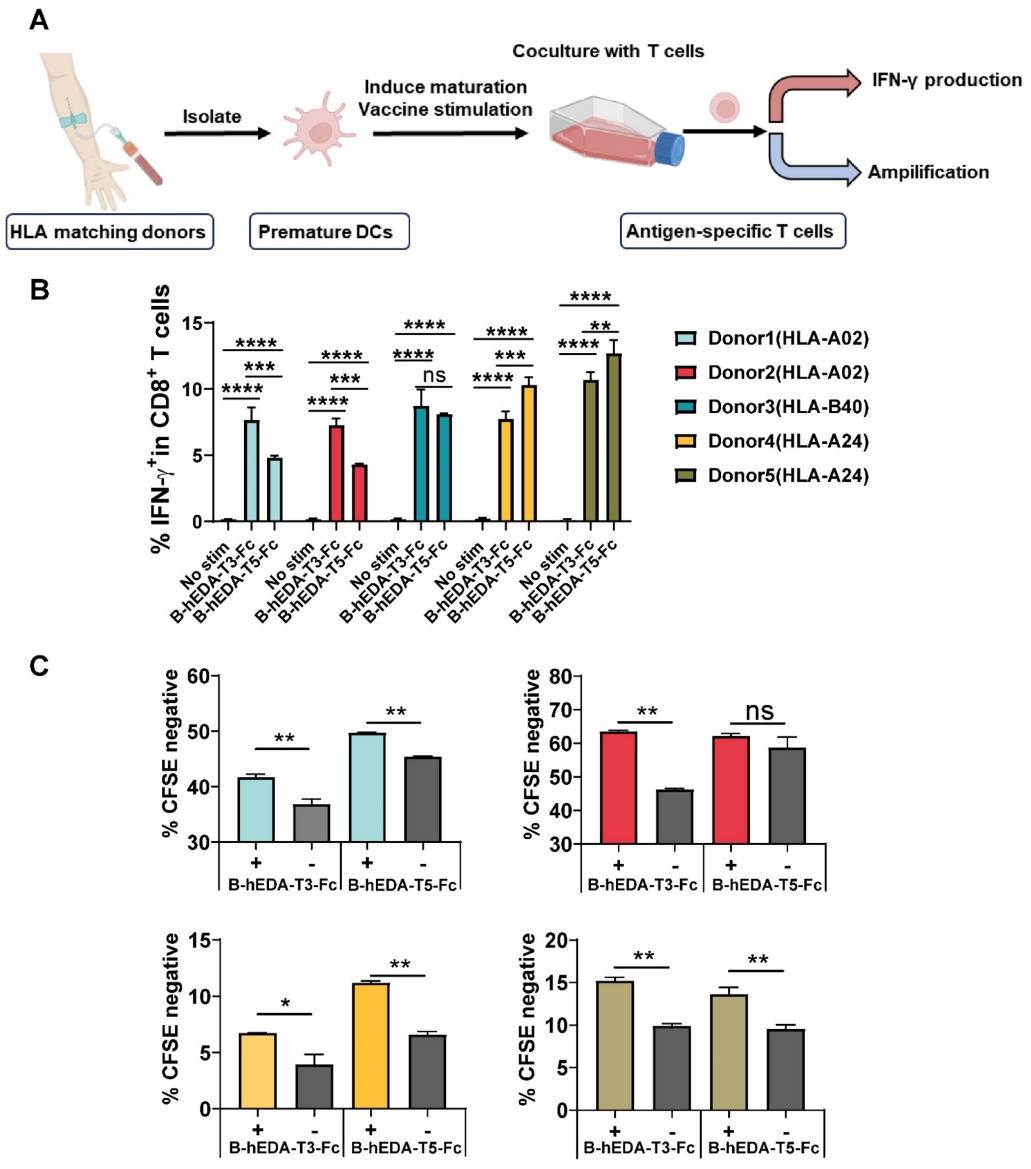
** Recombinant vaccines induced HLA isotype-dependent T-cell activation and proliferation**. **A**) Schematic representation of the DC-T-cell coculture assay. PBMCs from HLA-matched donors were isolated, and DCs were separated from PBMCs and induced to mature. Recombinant vaccines were added during DC maturation. Subsequently, autogenous T cells were cocultured with mature DCs for 9 days, and IFN-γ production and T-cell amplification were assessed. **B**) Summary of IFN-γ production by CD8^+^ T cells from 5 healthy donors after stimulation with different vaccines, with no stim as no stimulation. **C**) T-cell amplification was assessed with the cell division tracker CFSE using flow cytometry, and the quantification of T-cell amplification is shown, with each group subjected to a replication test. The data are presented as the mean ± SD. Statistical significance was calculated using two-way ANOVA with Tukey's multiple comparisons test, and significance was designated when the p value was less than 0.05 (* p < 0.05, ** p < 0.01, *** p < 0.001, **** p < 0.0001).

Table S4 presents data on six EERs matched with different HLA-I and HLA-II isotypes, with the most prevalent being HLA-A02, HLA-A24 and HLA-B40. To investigate whether recombinant vaccines could trigger the activation and proliferation of T cells specific to the corresponding HLA isotype, we conducted a coculture assay involving human dendritic cells (DCs) and T cells 45, as outlined in Figure 2A. Five healthy donors with identified HLAs were selected, and DCs were isolated and cocultured with their respective T cells (Table S5). To verify DC maturation postvaccine incubation, we collected DCs at two time points and assessed CD86 expression levels, which were found to increase after induction (Figure S3A-B). After 9 days of coculture, the T cells were stimulated with each vaccine for 24 h, and the production of IFN-γ was quantified by flow cytometry. A significant increase in IFN-γ production was observed in all groups compared to that in the unstimulated group (Figure S4A-B). However, when comparing B-hEDA-T3-Fc and B-hEDA-T5-Fc, an HLA allele-dependent difference was noted among the four donors. Specifically, compared to B-hEDA-T5-Fc, B-hEDA-T3-Fc, which contained more HLA-A02 matching epitopes, exhibited superior T-cell stimulation in donor 1 and donor 2, whereas B-hEDA-T5-Fc, with more HLA-A24 matching epitopes, preferentially activated more T cells in donor 4 and donor 5 than did B-hEDA-T3-Fc. No difference in IFN-γ production was observed in HLA-B40^+^ donor 3 (Figure 2B), which was attributed to the shared HLA-B40-matched epitope in EER 6 in both vaccine designs. Additionally, we labeled T cells with a cell division tracker, carboxyfluorescein succinimidyl ester (CFSE), and observed a noticeable decrease in CFSE intensity after stimulation by the respective vaccine-loaded DCs (Figure 2C and Figure S5). Thus, the vaccines we designed effectively activate T cells after antigen presentation by DCs from donors with corresponding HLA isotypes, thereby eliciting specific cellular immune responses.

### B-hEDA-T3-Fc and B-hEDA-T5-Fc show differential antitumor activity in a preventive model

**Figure 3:**
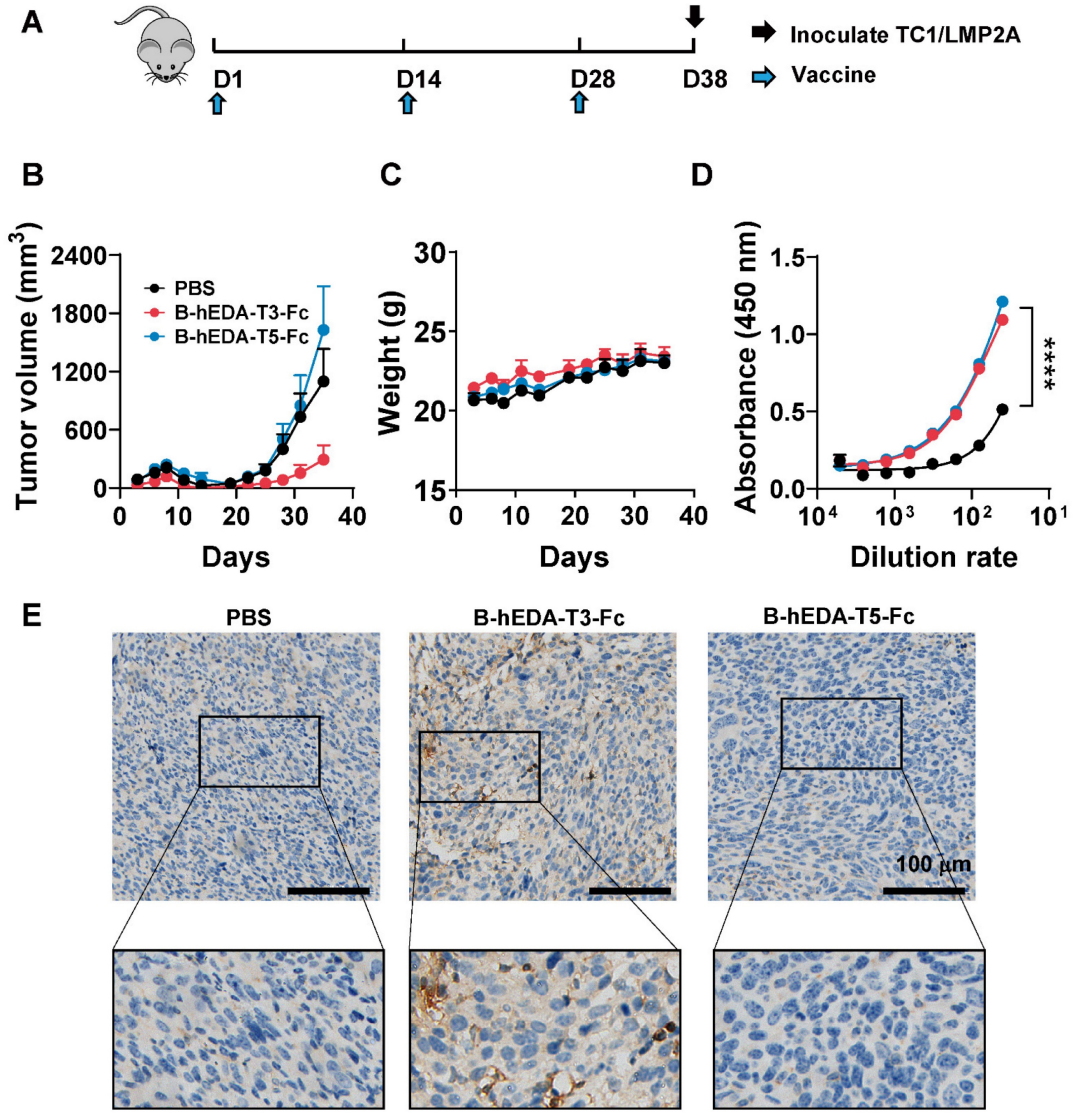
** Differential antitumor activity of B-hEDA-T3-Fc and B-hEDA-T5-Fc in the TC1/LMP2A preventive model. A**) Overview of the TC1/LMP2A preventive assay procedure. C57BL/6J mice (n = 5) were subcutaneously immunized with 1 nmol of B-hEDA-T3-Fc or B-hEDA-TEC5-Fc and boosted with the same dose on days 14 and 28. PBS was administered as a control. Ten days after the last immunization, 2 × 10^6^ TC1/LMP2A cells were inoculated into the right flank of the mice. **B**) Monitoring of tumor volume until the volume reached 2000 mm^3^ or severe ulceration of tumor tissue. Tumor volume was calculated as W × L × H and measured using digital calipers. Each data point represents the mean from 5 mice per group ± SD. **C**) Mouse body weight in the TC1/LMP2A preventive model (n = 5). **D)** Serum samples from each group were collected 7 days after the last immunization, and the serum antibody titers were assessed using TC1/LMP2A cell-based ELISA. Each dilution was tested in duplicate experiments (n = 3). **E**) Tumor tissues were collected at the endpoint of the experiment, and immunohistochemistry was performed to detect the infiltration of CD3^+^ T cells in tumor tissues. Scale bars are shown, indicating 100 μm. The marked area was amplified to illustrate the infiltration of CD3^+^ T cells. Statistical significance was calculated using two-way ANOVA with Tukey's multiple comparisons test, and significance was designated when the p value was less than 0.05(* p < 0.05, ** p < 0.01, *** p < 0.001, **** p < 0.0001).

To evaluate the preventive efficacy of the recombinant vaccines, we established two mouse tumor cell lines expressing LMP2A, designated TC1/LMP2A and MC38/LMP2A (Figure S6A-B), and confirmed LMP2A expression via reverse transcription PCR (Figure S6C-D). Subsequently, we assessed the prophylactic effectiveness of these vaccines using the TC1/LMP2A cell line. Briefly, C57BL/6J mice received subcutaneous injections of 1 nmol/dose of each vaccine every two weeks for three cycles, followed by inoculation with 2 × 10^6^ TC1/LMP2A cells ten days later (Figure 3A). Remarkably, mice vaccinated with B-hEDA-T3-Fc exhibited significant tumor regression, whereas no inhibition of tumor growth was observed in the mice immunized with B-hEDA-T5-Fc (Figure 3B-C). Despite similar serum antibody titers between mice inoculated with B-hEDA-T5-Fc and B-hEDA-T3-Fc (Figure 3D), which could be attributed to the shared B-cell epitope (199-209) of both vaccines, greater infiltration of T cells into tumor tissues was noted in the B-hEDA-T3-Fc group than in the B-hEDA-T5-Fc group (Figure 3E). Considering the distinct MHC isotypes between humans and mice, we conducted epitope prediction with C57BL/6J MHC H-2Db for B, T3 and T5, which confirmed that the binding affinity of T5 and B to H-2Db was significantly weaker than that of T3 (Table S6), leading to unsatisfactory tumor inhibition efficacy.

**Figure 4:**
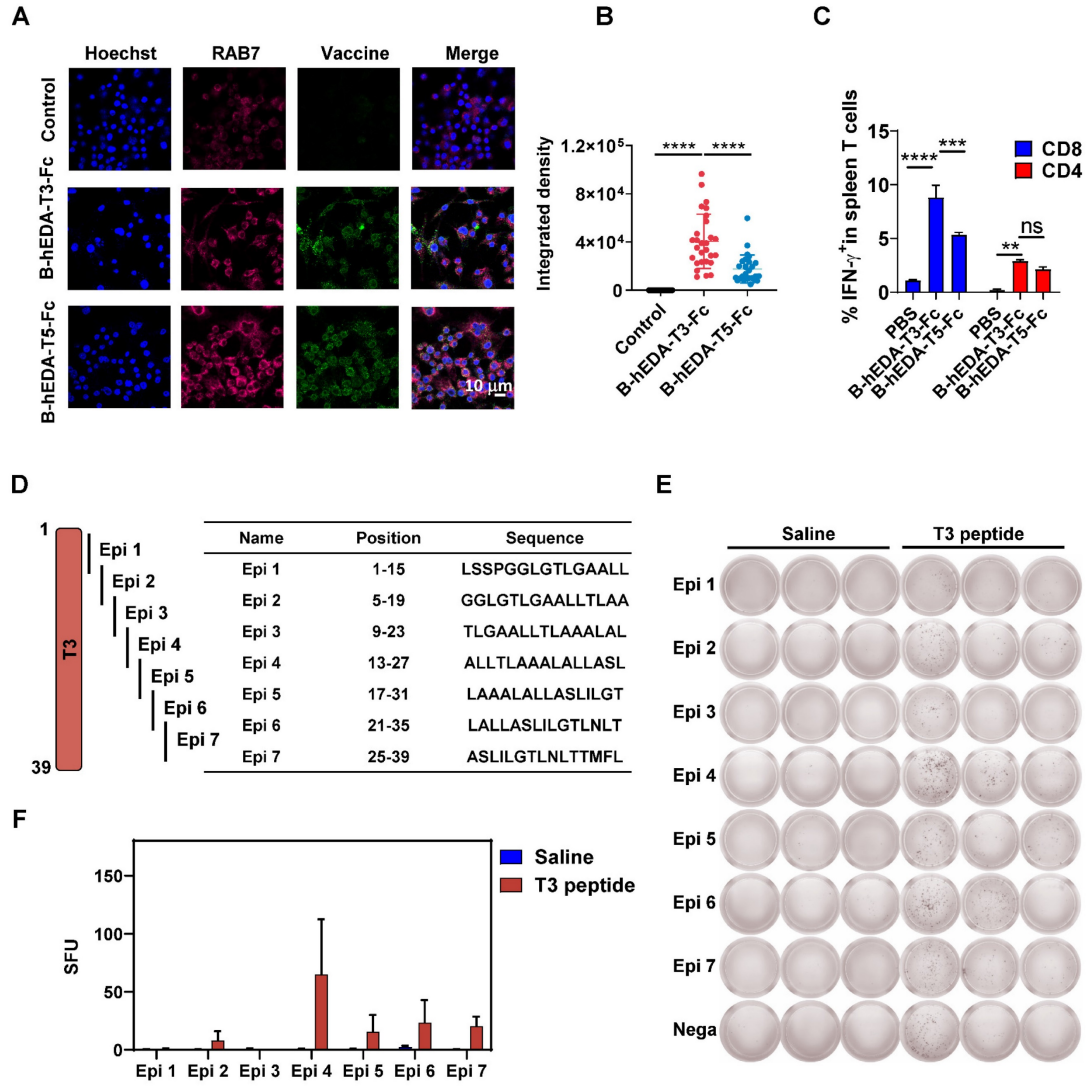
** Induction of cellular immunity by B-hEDA-T3-Fc and B-hEDA-T5-Fc and the validation of functional epitopes**. **A**) Colocalization of the recombinant vaccines with lysosomes was examined by incubating RAW264.7 cells with 100 nM vaccine for 1 h. Immunofluorescence staining was performed using the corresponding antibodies (magenta: AF647-anti-RAB7; blue: Hoechst; green: AF488-anti-Flag). The scale bar in the last picture indicates 10 μm. **B**) Quantification of the colocalization of the integrated fluorescence density is shown (n = 28). **C**) Antigen-specific T cells were detected via intracellular cytokine staining. Female C57BL/6J mice (n = 3) were immunized three times at two-week intervals. Ten days after the last immunization, the splenocytes were collected and stimulated with the corresponding vaccine at 10 μg/mL for 24 h with the addition of 2 μM brefeldin A during the last 5 h, and IFN-γ was detected using flow cytometry. Statistical analysis of IFN-γ^+^ T cells after the last immunization with the vaccines (n = 3). **D**) Illustration and sequence summary of synthetic epitopes in T3. **E**) ELISpot results after stimulation with Epi 1 to Epi 7 (n = 3), with nega as the negative control. **F**) Statistical analysis of spot forming units (SFU) in E. The data are presented as the mean ± SD. Statistical significance was calculated using two-way ANOVA with Tukey's multiple comparisons test, and significance was designated when the p value was less than 0.05 (* p < 0.05, ** p < 0.01, *** p < 0.001, **** p < 0.0001).

To corroborate the differences in antitumor activity, we analyzed the antigen uptake efficiency of both vaccines in the mouse macrophage line RAW264.7 using immunofluorescence staining. By assessing the colocalization of RAB7 with each vaccine inside the cells 46, we observed greater uptake of B-hEDA-T3-Fc by RAW264.7 cells than of B-hEDA-T5-Fc (Figure 4A-B), suggesting increased digestion of B-hEDA-T3-Fc in lysosomes and, consequently, greater presentation of epitopes by MHC H-2Db. We speculated that the sequence and structural differences (Figure S2) contributed to the variance in antigen presentation between these vaccines, and colocalization outcomes aligned with the predicted results (Table S6). Furthermore, analysis of the cellular immune response of T cells from vaccinated mice revealed that B-hEDA-T3-Fc induced a greater proportion of IFN-γ^+^ CD8^+^ T cells than did B-hEDA-T5-Fc, with no significant difference in the proportion of CD4^+^ T cells (Figure 4C and Figure S7A). Consistently, ELISpot assay results confirmed that splenocytes from the B-hEDA-T3-Fc group exhibited more spots, indicating IFN-γ secretion, after vaccine stimulation than those from the B-hEDA-T5-Fc group (Figure S7B-C). The efficacy of B-hEDA-T3-Fc was further assessed in an MC38/LMP2A preventive model via a procedure mirroring that in the TC1/LMP2A preventive assay. Significant inhibition of tumor progression was observed in the B-hEDA-T3-Fc-vaccinated mice, which exhibited 5/5 complete responses (Figure S8A-B). Overall, our findings validate that recombinant vaccines prepared through epitope prediction and splicing elicit specific T-cell responses aligned with MHC isotype dependence, underscoring the feasibility and practicality of our design strategy.

We hypothesized that the variation in tumor inhibition could be attributed to differences in T-cell EERs. To identify the functional epitopes within the EER3 region, we synthesized the complete set of EER3 peptides and divided the sequence into seven smaller peptides, designated Epi 1 to Epi 7. Each peptide was 15 amino acids in length, with an 11-amino acid overlap between adjacent peptides to ensure comprehensive sequence coverage (Figure 4D). Mice (n = 3) were subcutaneously immunized with 50 μg of the T3 peptide four times at two-week intervals. Three days after the final immunization, splenocytes were harvested and subjected to IFN-γ ELISpot analysis. Epi 1 to Epi 7 were then used as stimuli to evaluate IFN-γ responses. Our results showed that splenocytes from T3-immunized mice generated significantly more IFN-γ spots when stimulated with Epi 2, Epi 4, Epi 5, Epi 6, and Epi 7, suggesting these five epitopes are functional within the T3 EER region (Figures 4E-F). Among them, peptide Epi 4 (ALLTLAAALALLASL) stood out as the most immunogenic, exhibiting the highest capacity to elicit robust CD8^+^ T-cell responses, suggesting its potential as a critical component for driving potent antigen-specific immunity.

### B-hEDA-T3-Fc demonstrates superior antitumor efficacy compared to split vaccines

**Figure 5:**
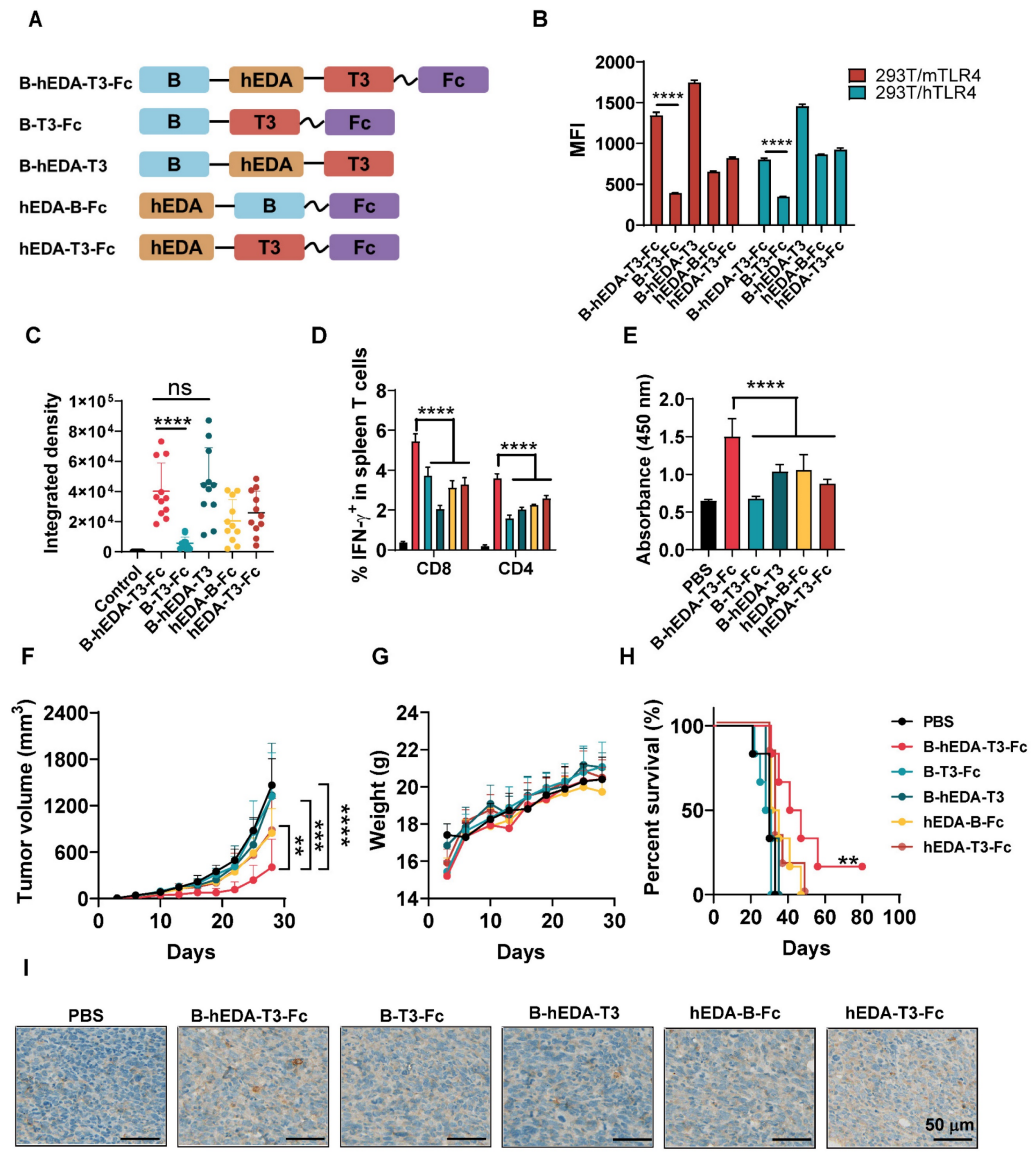
** The antitumor activity of B-hEDA-T3-Fc was greater than that of split-component vaccines**. **A**) Schematic of the design of B-hEDA-T3-Fc split-component vaccines. **B**) TLR4 binding affinity of B-hEDA-T3-Fc and its split-component vaccines. **C**) 0.5 × 10^6^ RAW264.7 cells were cultured on slides for 24 h before 100 nM vaccines addition, after 1 h culture, cells were fixed, permeabilized and stained, quantification of fluorescent integrated density (n = 11) is shown. **D**) Analysis of cellular immunity through examination of vaccine-specific IFN-γ^+^ T cells among isolated mouse splenocytes (n = 3) treated with the indicated vaccines. The mice were immunized with an equimolar dose of 1 nmol in each group. Splenocytes were collected seven days after the third vaccination, and the quantification of IFN-γ^+^ T cells in the splenocyte population is shown. **E**) Serum antibody titer results at a 1:20 dilution. The data were separated and are displayed accordingly. **F**) Tumor volume and **G**) body weights were monitored twice per week in the MC38/LMP2A therapeutic model (n = 6). **H**) Kaplan-Meier curves illustrating the overall survival of mice (n = 6) treated with the indicated vaccines. **I**) Infiltration of mouse CD3^+^ cells in MC38/LMP2A tumor tissue. Representative images are shown, with scale bars indicating 50 μm. The data are presented as the mean ± SD. Statistical significance was calculated using two-way ANOVA with Tukey's multiple comparisons test, and significance was designated when the p value was less than 0.05 (* p < 0.05, ** p < 0.01, *** p < 0.001, **** p < 0.0001).

To identify the pivotal component of B-hEDA-T3-Fc responsible for tumor inhibition, we designed four split-component candidates derived from B-hEDA-T3-Fc, designated B-T3-Fc, B-hEDA-T3, hEDA-B-Fc and hEDA-T3-Fc (Figure 5A). Electrophoresis results confirmed that their purity exceeded 90%, with migration at the exact molecular weight (Figure S9). The necessity of hEDA fusion was further validated through TLR4 binding and activation studies. Two TLR4-expressing 293T cell lines, 293T/mTLR4 and 293T/hTLR4, were successfully established (Figure S10A-B). Using flow cytometry, we evaluated the biding affinity of B-hEDA-T3-Fc and its split component variants (Figures 5B and Figure S10C). The results demonstrated that hEDA effectively bound both hTLR4 and mTLR4, with binding efficiency influenced by its position within the vaccine constructs. When hEDA was placed between the two EER regions, it displayed higher affinity for TLR4 compared to its placement at the N-terminus. Among the constructs, B-hEDA-T3 exhibited the strongest binding affinity, likely due to reduced polymerization from the absence of the Fc fragment, enhancing its binding efficiency. To evaluate downstream activation from the hEDA-TLR4 interaction, we assessed NF-κB signaling and TNF-α secretion in THP1 cells (Figure S10D). The incorporation of hEDA significantly enhanced TNF-α secretion, confirming its role in activating innate immune pathways. We also examined vaccine uptake by antigen-presenting cells to analyze the colocalization of the vaccine and RAB7, which revealed augmented uptake and digestion for all candidates except B-T3-Fc, which lacks hEDA, emphasizing its role in facilitating efficient vaccine capture (Figure 5C and Figure S11). Next, we compared the cellular immunity elicited by the different vaccines.

C57BL/6J mice were immunized one day after tumor inoculation and boosted with two additional shots at a 5-day interval. Seven days after the final immunization, splenocytes were collected and stimulated with the corresponding vaccines. As shown in Figures 5D and Figure S12, significantly fewer IFN-γ^+^ CD8^+^ and CD4^+^ T cells were detected in the split-component vaccine groups than in the B-hEDA-T3-Fc group. Notably, compared with B-hEDA-T3-Fc, hEDA-B-Fc lacking T3 exhibited relatively low T-cell activation, highlighting the importance of the T3 component for triggering an enhanced cellular immune response. In addition to cellular immunity, we examined humoral immune function by immunizing mice three times and assessing serum antibody titers via MC38/LMP2A cell- based ELISA. As illustrated in Figures 5E and Figure S13, B-hEDA-T3-Fc induced greater humoral immunity than did the split-component candidates, and the removal of different components significantly affected antibody titers. Notably, mice immunized with B-T3-Fc lacking hEDA showed nearly undetectable levels of LMP2A^+^ tumor cell-specific antibodies, in contrast to those in the PBS group. This result highlights the necessity of integrating diverse components into designed vaccines that target both innate and adoptive immunity, with hEDA playing a pivotal role in antigen uptake and cross-presentation, thereby promoting efficient activation and differentiation of both B and T cells.

To verify the antitumor efficacy of B-hEDA-T3-Fc, we evaluated its activity in an MC38/LMP2A therapeutic model. As depicted in Figure 5F, significant tumor growth inhibition was observed with B-hEDA-T3-Fc treatment on day 28, whereas mice treated with the split-component candidates showed tumor recurrence soon after immunization. Importantly, B-hEDA-T3-Fc-treated mice did not exhibit severe body weight loss compared to those in the split-component vaccine groups (Figure 5G). Consistent with the *in vivo* efficacy, mice treated with B-hEDA-T3-Fc showed significantly prolonged survival (Figure 5H). Enhanced T-cell infiltration into tumor tissues was also observed in mice immunized with B-hEDA-T3-Fc compared to that in mice immunized with other candidates (Figure 5I). These results underscore the efficacy of our unique B-hEDA-T3-Fc design for enhancing antigen presentation and inducing robust humoral and cellular immunity, effectively inhibiting and overcoming tumor relapse associated with traditional vaccines lacking essential components.

### Synergistic antitumor activity of B-hEDA-T3-Fc and a PD-1 inhibitor

**Figure 6:**
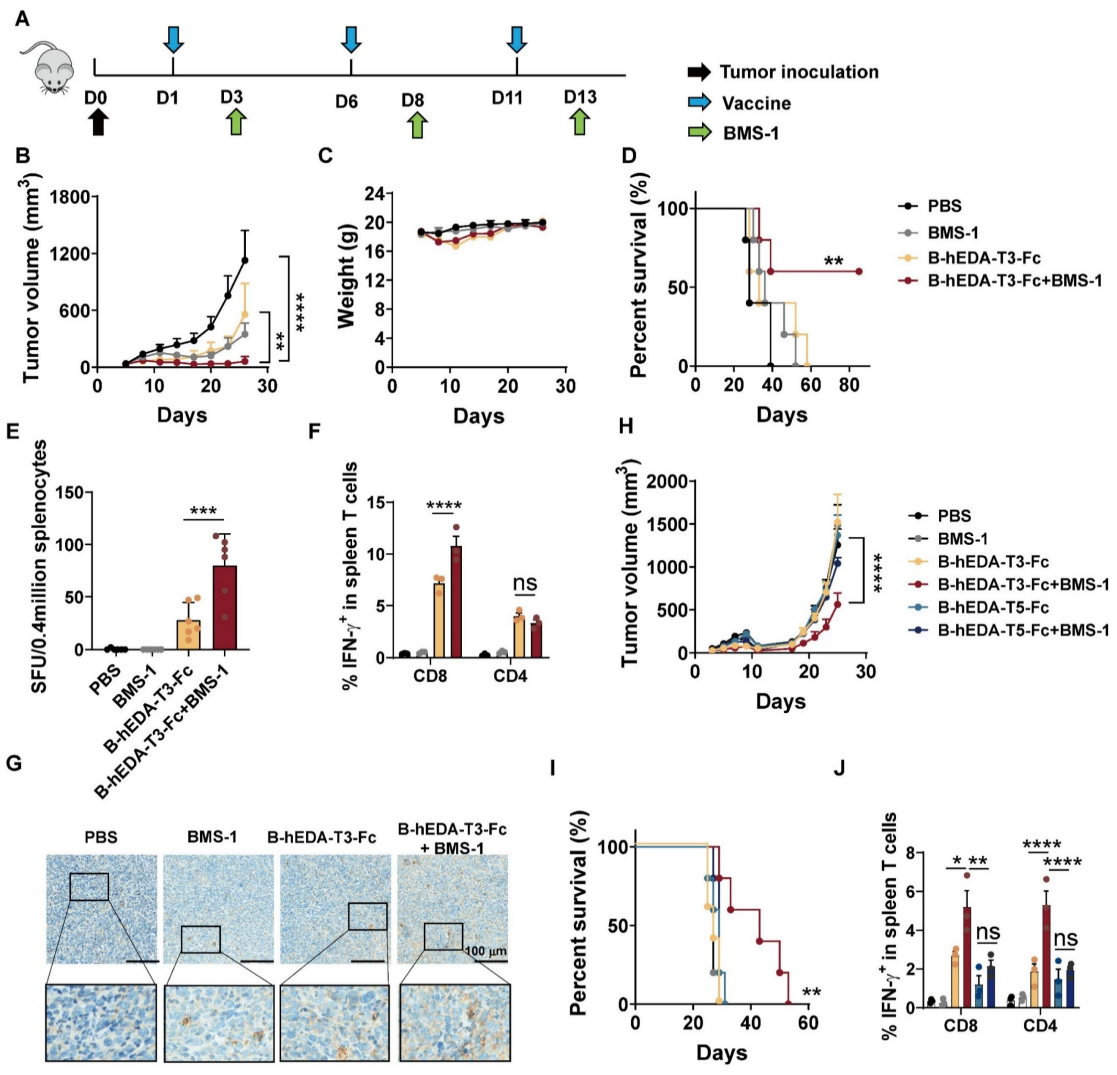
** Synergistic antitumor activities of recombinant vaccines in combination with PD-1 inhibitors in therapeutic models**. **A**) Illustration of the therapeutic assay schedule. A total of 2 × 10^6^ TC1/LMP2A or 1 × 10^6^ MC38/LMP2A cells were inoculated into the right flank of six-week-old C57BL/6J mice (n = 5). Subsequently, 1 nmol of recombinant vaccine was administered subcutaneously the following day, with two additional doses given at five-day intervals. Two days after each immunization, 100 μL of BMS-1 (10 μg/mL) was administered intraperitoneally. **B**) Monitoring of tumor volume and **C**) body weight during the MC38/LMP2A therapeutic experiment. **D**) Kaplan-Meier curves showing the overall survival of mice treated as indicated in (A). The data were analyzed using the log-rank test. **E**) IFN-γ ELISpot analysis of isolated splenocytes seven days after the last vaccination (n = 3) in the MC38/LMP2A therapeutic assay. **F**) Quantification of IFN-γ^+^ T cells in the splenocyte population (n = 3). Splenocytes were collected 7 days after the last immunization and stimulated with B-hEDA-T3-Fc to analyze the number of spot-forming unit. **G**) Infiltration of mouse CD3^+^ cells in MC38/LMP2A tumor tissue. Representative images are shown, with scale bars indicating 100 μm. **H**) Monitoring tumor volume and **I**) survival in TC1/LMP2A therapeutic assays (n = 5). **J**) Summary of the number of IFN-γ^+^ T cells among splenocytes in the TC1/LMP2A therapeutic model (n = 3). The data are presented as the mean ± SD. Statistical significance was calculated using two-way ANOVA with Tukey's multiple comparisons test, and significance was designated when the p value was less than 0.05(* p < 0.05, ** p < 0.01, *** p < 0.001, **** p < 0.0001).

Combining cancer vaccines with PD-1 inhibitors holds promise for treating solid tumors by leveraging the natural immune response boosting by PD-1 blockade. To explore the long-term therapeutic efficacy in the MC38/LMP2A model, we evaluated antitumor efficacy by combining the vaccine with the PD-1 inhibitor BMS-1 47, 48, as outlined in Figure 6A. While both B-hEDA-T3-Fc and BMS-1 alone partially alleviated the tumor burden, the combination treatment persistently significantly inhibited tumor growth (Figure 6B-6C). Correspondingly, this combination therapy led to markedly prolonged survival compared to that with monotherapy with either the vaccine or PD-1 inhibitor (Figure 6D). In parallel, splenocytes were collected for ELISpot and intracellular cytokine detection. Compared with those receiving other treatments, mice receiving both B-hEDA-T3-Fc and BMS-1 showed increased numbers of IFN-γ-producing T cells (Figure 6E). Further flow cytometry analysis revealed that the PD-1 inhibitor primarily enhanced the activation and proliferation of CD8^+^ T cells but not CD4^+^ T cells following vaccination (Figure 6F and Figure S14). Immunohistochemical staining of tumor tissues revealed a considerably greater number of infiltrating CD3^+^ T cells in the combination therapy group than in the monotherapy groups (Figure 6G).

Given the limited impact of post-tumorigenesis vaccination, particularly in lung cancer, which has a relatively 'cold' tumor phenotype, we assessed vaccine activity by co-administering B-hEDA-T3-Fc and BMS-1 in a TC1/LMP2A therapeutic model. As shown in Figure 6H, TC1/LMP2A tumor growth remained aggressive in the presence of either B-hEDA-T3-Fc or BMS-1 alone; however, significant tumor regression was observed in the combination group. Importantly, this synergistic effect was not observed in mice treated with BMS-1 alongside another vaccine, B-hEDA-T5-Fc, whose T5 component could not effectively induce a cellular immune response due to its weak binding affinity with H-2Db (Table S6). Compared with other regimens, the combination of B-hEDA-T3-Fc and BMS-1 significantly extended the survival of mice bearing TC1/LMP2A tumors (Figure 6I). To elucidate the underlying mechanism, splenocytes were collected to assess IFN-γ^+^ T cells after vaccination, revealing that the addition of BMS-1 rescued the proportion of CD8^+^ or CD4^+^ T cells from the inhibitory microenvironment (Figure 6J and Figure S15). However, in mice in which there were insufficient antigen-specific CD8^+^ T cells, as observed in the group treated with B-hEDA-T5-Fc, the addition of BMS-1 did not enhance T-cell activation. Further immunohistochemical staining results confirmed that BMS-1 efficiently activated and induced the proliferation of T cells in the tumor microenvironment, particularly in the context of vaccine epitopes matching MHC alleles (Figure S16). Taken together, these findings suggest that personalized vaccines with MHC allele dependence can exhibit synergistic activity with PD-1 blockade against tumors, whether immunogenic or not, resulting in systemic tumor regression and an overall improved therapeutic outcome.

## Discussion

Recombinant vaccine therapy has emerged as a promising clinical approach for patients with malignancies or infectious diseases. However, the inability to incorporate multifaceted functionality into vaccines has resulted in poor therapeutic efficacy and persistence. To address these limitations, several groups have explored combining PRR agonists or protein slow-release strategies to enhance vaccine activities 49-51. To our knowledge, there are no representative studies on the design and development of recombinant vaccines that integrate multifaceted approaches including* in silico* epitope prediction, EER splicing, and fusion with TLR agonists and long-acting molecules. Importantly, this study systemically describes the significance of optimizing the design of recombinant vaccines to achieve multipronged immune responses, compared to those induced by traditional designed candidates.

Building on the understanding of endogenous immunity, epitope prediction-assisted bioinformatic analysis has been increasingly explored in the design of various vaccines, particularly in response to the coronavirus disease 2019 pandemic 52, 53. Epitope prediction involves two main components, B-cell epitope prediction and T-cell epitope prediction, and among the numerous available prediction algorithms, the IEDB is the most comprehensive platform for epitope prediction and analysis 54. With the assistance of the IEDB, we identified prevalent HLA subtypes in Southeast Asia to predict LMP2A epitopes and evaluated these predictions using multiple criteria. Specifically, we utilized various tools, such as VaxiJen to assess epitope immunogenicity, ProtParam to analyze hydrophobicity, AllerTOP to evaluate allergenicity, and Toxinpred to predict toxicity. Through a rigorous candidate selection process, we identified 33 CTL epitopes and 16 HTL epitopes. Additionally, by incorporating the reported B-cell epitope (199-209), we spliced adjacent and overlapping epitopes to create six EERs, ensuring an epitope density exceeding 0.1 per amino acid length. Our predictions revealed differences between T3 and T5 in HLA-I isotypes, with T5 containing a unique HLA-A24-matching epitope, while T3 encompassed more HLA-A2-matching epitopes. Subsequently, DCs loaded with B-hEDA-T3-Fc and B-hEDA-T5-Fc stimulated IFN-γ production by human T cells, which correlated with donor HLA isotypes. Notably, similar personalized efficacy of recombinant vaccines was observed in mouse models. Compared to B-hEDA-T3-Fc, B-hEDA-T5-Fc more poorly inhibited the growth of TC1/LMP2A tumors in C57BL/6J mice due to the weaker binding ability of epitopes derived from B-hEDA-T5-Fc with the MHC allele H-2Db. In summary, this study underscores the critical role of epitope prediction in vaccine design, which directly impacts therapeutic activities across individuals with diverse genetic backgrounds.

Epitope-based vaccines, also known as peptide-based vaccines, have demonstrated significant clinical benefits via activation of T cells or B cells in response to pathogens 55, 56. However, the traditional approach using truncated 8-17 amino acid fragments may disrupt spatial epitopes, particularly discontinuous fragments. Efforts have been made to improve upon this design by fusing epitopes together via three common methods: 1) direct fusion of multiple epitopes, 2) separation of epitopes with protease cleavage sites, and 3) separation of epitopes with noncleaved linkers 21, 44, 57. In our study, we analyzed and spliced high epitope density regions in natural antigen sequences to form six separate EERs, each containing 4 to 7 epitopes with lengths ranging from 26 to 48 amino acids, based on our prediction results. We then selected EER 6, which contains a B-cell epitope, and either EER 3 or 5, which exhibit structures representative of T-cell epitope-rich regions that are MHC allele dependent, to ensure epitope diversity of the target antigen LMP2A. While EER-based vaccines have been tested previously 44, 58, a common drawback among these approaches is severe aggregation. To address this issue, we optimized the design of spliced EERs with TLR agonists and the IgG1 Fc region by carefully selecting their positions and linkers. Instead of directly combining different EERs, we introduced the hydrophilic hEDA between the hydrophobic EER 6 and EER 3 or 5, followed by the hydrophilic Fc part, adopting a hydrophobic-hydrophilic-hydrophobic-hydrophilic format to enhance the stability and solubility of the recombinant vaccines. We used a rigid EAAAK linker to connect each EER with hEDA to ensure the full display of both EERs and reduce interference between them and a flexible GGGGS linker in front of the Fc portion to prevent disruption of Fc polymerization 59, 60. The prevalent formulation of vaccines involves the encapsulation of antigens with nanoparticles or hydrogel-based biocompatible materials, along with TLR and STING agonists 35, 36, 49, 50, where all the elements are aggregated by electrostatic interactions or hydrogen bonds; however, this multicomponent formulation of vaccines causes manufacturing and quality control difficulties. Our established EER splicing strategy, in which all elements are integrated into a rationally designed single molecule, ensures the necessary functionality of B-cell and T-cell epitopes along with immune enhancement, resulting in a multifaceted vaccine that induces significantly improved humoral and cellular immunity in both prevention and therapeutic tumor models.

As mentioned previously, vaccines can be combined with TLR agonists to enhance the immune response *in vivo* 61, 62. Activation of TLR4 is particularly promising because it stimulates robust antigen uptake and Th1 immune responses 63. TLR4 is expressed on the cell surface of DCs, monocytes, macrophages, T cells and B cells, and it recognizes various ligands, including lipopolysaccharide (LPS), LPS analogs and natural ligands such as hEDA and the respiratory syncytial virus fusion protein 64. Among these, hEDA is best suited for vaccine fusion because it activates TLR4 and improves the solubility of the fusion protein, which is crucial for insoluble target proteins, such as the multipass transmembrane protein LMP2A. Previous research has shown that hEDA upregulates DC maturation markers, such as CD80 and CD86, and that mice immunized with an hEDA-fused vaccine exhibit strong and long-lasting antigen-specific immune responses 27, 65. Our study comparing B-hEDA-T3-Fc with its split-component candidates indicated that the absence of hEDA significantly affects antigen uptake and digestion in antigen-presenting cells, thereby reducing humoral and cellular immune responses and compromising antitumor efficacy.

The establishment of sustained and long-term immune cell activation via vaccines is crucial for providing enduring protection against pathogens. Two primary approaches are commonly employed to prolong the half-life of vaccines: fusion with long-acting molecules or encapsulation of antigens in biocompatible materials. In our study, we fused the human IgG1 Fc fragment with EERs in the polymerization structure to extend the half-life, leveraging the ability of IgG1 to rescue proteins from endosomal degradation by binding to the neonatal Fc receptor (FcRn), thus facilitating protein recycling 66. Despite being expressed in prokaryotic cells, aglycoslated Fc did not exhibit impaired a pH-dependent interaction with FcRn *in vitro* or reduced serum persistence *in vivo* 67. Designing vaccines with the Fc portion incorporated holds promise for improving efficacy and protection persistence, potentially resulting in enhanced antitumor breadth and potency. Notably, our long-lasting vaccine induced robust humoral and cellular immune responses in therapeutic models and showed synergistic inhibition of tumor growth when combined with immune checkpoint inhibitors, suggesting promising clinical applications for patients with a poor prognosis due to tumor relapse or resistance to conventional PD-1 therapy.

The initial goal of this study was to develop multifunctional vaccines tailored for individuals with EBV-associated NPC in Southeast Asia, aiming to induce targeted T-cell immune responses in populations with specific HLA subtypes. While B-hEDA-T3-Fc and B-hEDA-T5-Fc effectively stimulated the activation and proliferation of T cells from donors with HLA-A02 and HLA-A24, respectively, the specificity of these vaccines in mouse models could not be confirmed due to the disparity between human and mouse MHC. To address this limitation, the utilization of transgenic mice, specifically HLA transgenic mice, for testing these vaccines will clearly elucidate the MHC-dependent mechanisms underlying epitope-oriented vaccines 21, 68. This animal study also highlights the additional challenge of establishing xenograft models of human NPC in immunocompetent mice. Although our optimally designed vaccines significantly inhibited the growth of LMP2A-transduced mouse tumors, practical application of human NPC cell lines in mouse models with reconstituted human immune systems is essential for determining the therapeutic efficacy of these vaccines in humans. While many LMP2A epitopes have been identified previously 69, 70, the efficacy of the T3 EER (288-326) remains underexplored. Our findings highlight the importance of EER3, expanding the repertoire of LMP2A epitopes and contributing valuable insights into the development of NPC-targeted vaccine.

## Conclusion

In conclusion, we established a platform for the precise design of vaccines that incorporate multiple immune-enhancing elements and successfully applied this approach to develop vaccines targeting LMP2A. Our findings indicate that epitope prediction-guided vaccine design is a promising and efficient method for antigen exploration, particularly when combined with an epitope splicing strategy and the integration of TLR agonists and long-acting molecules. Therefore, optimized LMP2A-targeted vaccines could serve as promising therapeutic options for NPC patients, and this recombination approach may have broader applications in vaccine design for targeting other types of cancers, as well as nononcology indications.

## Methods

### Epitope prediction and splicing

To predict LMP2A epitopes, we utilized online databases IEDB. Our methodology involved several key steps: Initially, we collected MHC frequency data from Southeast Asia, focusing specifically on populations from Hong Kong and South China. We referenced the Allele Frequency Net Database, extracting MHC I and MHC II frequencies for various populations, including China Guangdong Province (n = 403), China Guangdong Province Meizhou Han(n = 100), China Guangxi Region Maonan (n = 108), China Guangzhou (n = 102), China Guangzhou Han (n = 106), China Guangzhou Han pop 2 (n = 86), China Han Guangdong Province (n = 208), China Hong Kong and Singapore (n = 135), China South Han (n = 284), and China South Han pop 2 (n = 1098). HLA alleles with a distribution rate exceeding 10% were selected as prediction HLA alleles.

Subsequently, using the identified HLA alleles, we predicted LMP2A-EBVB9 epitopes, encompassing both CTL epitopes and HTL epitopes. For epitope evaluation, we utilized various tools: VaxiJen for predicting immunogenicity, AllerTOP v.2.0 for assessing allergenicity, and ToxinPred for toxicity prediction. Furthermore, epitope selection was refined based on multiple criteria: a) consistent high-ranking across all prediction results; b) likelihood of being an antigen; c) probable non-allergen nature, and d) non-toxic characteristics. Finally, we combined adjacent and overlapping epitopes to form extended epitope enrichment regions (EERs), ensuring a density of epitopes per amino acid exceeding 0.1 (epitope number/EER length > 0.1).

This comprehensive approach aimed to identify and refine potential epitopes, laying the groundwork for their consideration in the development of an effective LMP2A-targeting recombinant vaccine.

### Cell lines

The mouse lung cancer cell line TC1 was purchased from Xiamen Immocell Biotechnology Co., Ltd. and validated through STR profiling. The mouse colon cancer cell line MC38, and mouse macrophage cell line RAW264.7 were obtained from the American Type Culture Collection. Additionally, the human embryo kidney cell line 293FT and 293T were sourced from Thermo Fisher Scientific, and the lymphoblastoid marmoset cell line B95-8 was generously provided by Prof. Junmin Quan. For culturing, 293FT, 293T, TC1 and MC38 cells were maintained in Dulbecco modified Eagle medium (DMEM) (Gibco, C11965500BT), supplemented with 10% fetal calf serum, 1% antibiotics and 2 mM L-glutamine. RAW264.7 and B95-8 cells were cultured in Roswell Park Memorial Institute (RPMI)-1640 medium (Hyclone, SH30255.01), supplemented with 10% fetal calf serum, 1% antibiotics and 2 mM L-glutamine.

### Plasmid construction

All plasmids were meticulously constructed using homologous recombination in accordance with established guidelines. Specifically, six recombinant vaccines were integrated into the pET-32a expression vector. For instance, in the case of B-hEDA-T3-Fc, the recombinant sequence B-hEDA-T3 was synthesized by Genewiz, Inc (Suzhou, China). Subsequently, overlapping PCR was employed to fuse the IgG1 Fc fragment to the C-terminus of B-hEDA-T3. The resulting overlapping PCR product was then inserted between the MscI and XhoI restriction enzyme sites, following the tag region containing maltose binding protein (MBP), a thrombin cutting site, and a Flag tag. Similarly, B-hEDA-T5-Fc was constructed using the same methodology. For the construction of hEDA-T3-Fc and hEDA-B-Fc, overlapping PCR to utilized to amplify hEDA-B-Fc or hEDA-T3-Fc, and these fragments were subsequently inserted into the pET-32a vector between NdeI and XhoI, following the identical tag region. To construct B-hEDA-T3, a tag fragment comprising a His tag and a thrombin cleavage site was appended to the N-terminus of B-hEDA-T3 using overlapping PCR. The resulting sequence was then inserted into the pET-32a vector between NdeI and XhoI. To prevent unintended thrombin digestion in the Fc hinge region, a mutation was introduced at K559, replacing it with Alanine.

To establish LMP2A stable transduced cell lines, a lentiviral vector was prepared based on the pELPS vector. The LMP2A(B95-8)-P2A fragment was synthesized by Genewiz, Inc, with EGFP connected following a P2A linker. Subsequently, the synthesized LMP2A-P2A-EGFP fragment was inserted into the pELPS vector between BamHI and SalI sites. All plasmids underwent stringent verification through sequencing and enzyme digestion assays.

### Protein expression and purification

The expression of B-hEDA-T3-Fc, B-hEDA-T5-Fc, hEDA-T3-Fc, hEDA-B-Fc, and B-T3-Fc was initiated by transforming plasmids containing each vaccine into Rosetta gami *E.coli* competent cells via a 45 s heat shock at 42 ℃. The strains were cultured at 37 ℃ until the OD_600nm_ reached 0.8-1.0, followed by induction with 0.1 mM IPTG for 20 h at 18 ℃. The resulting cell pellet was collected by centrifugation at 4 ℃ for 10 min. Purification of these recombinant vaccines involved a two-round affinity chromatography process. After ultrasonication to lyse the pellet, the lysate was centrifuged to remove cell debris, and the supernatant was filtered using a 0.45 μm filter membrane. Dextrin Beads (Smart-lifesciences, SA077025) were employed in the first affinity chromatography step to bind proteins containing the MBP fragment. Prior to thrombin digestion, the protein solvent was exchanged to Tris-NaCl via dialysis. Thrombin cleavage was then performed at a ratio of 1 IU/mg proteins at room temperature for 20 h. Subsequently, the second-round affinity chromatography utilized Protein G (GenScript, L00209) to bind Fc-based vaccines, with protein elution performed with 0.1 M Gly-HCl (pH 2.0).

For the expression of B-hEDA-T3, Rosetta gami competent cells containing pET32a-His-B-hEDA-T3 were cultivated in 2YT culture at 37 ℃ until the OD_600nm_ reached 0.8-1.0. Subsequently, 1 mM IPTG was added to the culture, and the cells were further cultured at 37 ℃ for an additional 4 h. Due to the low solubility of B-hEDA-T3, a strategy of protein inclusion body refolding was employed for purification. The resulting pellet was suspended in Lysis buffer (50 mM Tris-HCl, 1 mM EDTA, 100 mM NaCl, pH 8.0) for ultrasonication, and the collected cell debris was washed with Wash buffer (50 mM Tris-HCl, 1 mM EDTA, 100 mM NaCl, Triton-X 100 0.5%, pH 8.0) until the pellet turned gray-white. Subsequently, the pellet was solubilized with fresh Solubilization buffer (50 mM Tris-HCl, 1 mM EDTA, 100 mM NaCl, 8 M urea), and the solvent was replaced with Tris-NaCl (pH 8.0) through dialysis.

### LC-MS analysis

Protein molecular weights were determined using LC-MS analysis. Initially, proteins were desalted with ddH_2_O in an ultrafiltration tube. Subsequently, 50 mM Dithiothreitol was added to the protein solution and incubated at 57 ℃ for 45 min to cleave the disulfide bonds in the Fc fragment. The solution was then transferred to an LC sample tube, and the molecular weight of each protein was detected using Q Exactive HF-X (Thermo Fisher Scientific). Data analysis was conducted using BioPharma Finder 3.2 software.

### Lentivirus production and transfection

Transfection of 293FT cells was conducted using Lipofactamine 2000 (Thermo Fisher Scientific, 2854765). Cells were transfected with the plasmid pELPS-LMP2A-P2A-EGFP along with three support plasmids (pMDL, pRSV-Rev, VSV-G) for 48 h. The lentivirus-containing supernatant was then harvested and used to infect TC1 and MC38 cells. Initially, the cell concentration was adjusted to 6 × 10^6^ cells/mL, and 50 μL of cells were infected with 0.9 mL of virus supernatant. Subsequently, 6 μg/mL of polybrene (MERCK, TR-1003-G) was added, and the cells were cultured for an additional 15 h. Following this, the supernatant was removed, and fresh DMEM medium was added. Single clones of TC1/LMP2A and MC38/LMP2A were sorted based on EGFP positivity. The generation of 293T/mTLR4 and 293T/hTLR4 cell lines followed the same procedure as that used for TC1/LMP2A or MC38/LMP2A.

### LMP2A detection

Single clones of TC1/LMP2A and MC38/LMP2A were assessed using reverse transcriptase PCR. Two specific primers were designed to amplify the target sequence of LMP2A: the forward primer sequence was 5'-ATGACTCATCTCAACACATA-3', and the reverse primer sequence was 5'-CATGTTAGGCAAATTGCAAA-3'. Additionally, two primers were designed for amplifying of glyceraldehyde-3-phosphate dehydrogenase (GAPDH): the forward primer sequence was 5'-GGTGGTCTCCTCTGACTTCAACA-3', and the reverse primer sequence was 5'-GTTGCTGTAGCCAAATTCGTTGT-3'. Monoclonal cells of TC1/LMP2A and MC38/LMP2A were cultivated in a 6-well plate. Upon reaching 80-90% confluency, the culture medium was aspirated, and 1 mL TRIzol (Invitrogen, 15596-026) was added to suspend the cells. Subsequently, 0.2 mL of chloroform (MERCK, 102445) was added, and the mixture was vortexed for 15 s until the color changed to pink. After a 2 min incubation, centrifugation was carried out at 14000 rpm, 4 ℃ for 10 min. The supernatant was collected, and 0.5 mL isopropanol (Damao Chemical Reagent Factory, 2351) was added. Following a 10 min incubation at room temperature, centrifugation was repeated at 4 ℃ for 10 min. The supernatant was discarded, and the cell pellet was collected, and dissolved in DEPC-treated water (Sangon Biotech, 7732-18-5). For reverse transcription, a 4 × gDNA wiper was used to digest genome DNA, and RT-mix (Vazyme, R323-01) was used to reverse transcribe mRNA, serving as the template for PCR. The target sequence of LMP2A and the housekeeping gene GAPDH were then amplified using forward and reverse primers, and electrophoresis was employed for detection.

### Flow cytometry

Detailed information regarding the flow cytometry antibodies utilized in this study can be found in the supplementary materials (Table S7). Cell concentration was adjusted to 1-5 × 10^6^/mL in FACS buffer (5% BSA, 0.2% NaN_3_). Cells were stained with the corresponding antibody for 1 h on ice or 30 min at room temperature, with a FACS volume of 100 μL, unless otherwise specified. The antibody was diluted at a ratio of 1:100 in FACS buffer, resulting in a final concentration of 1-5 μg/mL. Following incubation with the fluorescently labeled antibodies, cells were washed with 1 mL FACS buffer, centrifuged at 12000 rpm for 1 min at room temperature. Subsequently, cells were resuspended in an appropriate volume of FACS buffer to achieve a concentration of 1 × 10^6^ cells/mL, and the signal was detected using ThermoFisher Attune NxT (Thermo Fisher Scientific, A29001). For cells requiring staining with multiple antibodies, various antibodies were mixed in one tube under conditions where the fluorescence from one antibody did not interfere with others. In case where interference occurred, compensation was performed. Data analysis was conducted using Flowjo_V10 software.

### Immunofluorescence observation

Glass slides were coated with 50 μg/mL poly-D-lysine (Sigma-Aldrich, 27964-99-4) overnight at 4 ℃. The coated slides were then placed in a 12-well plate, and 0.5 × 10^6^ RAW264.7 cells were cultured on the coated slides for 24 h. Subsequently, 100 nM vaccines were added to each well, and the cells were cultured for an additional 1 h. The cells were fixed with 4% paraformaldehyde (Biosharp, BL539A) for 10 min at room temperature, followed by washing with cold PBS three times. Cell permeabilization was achieved with PBS containing 0.25%Triton X-100 (Sangon Biotech, 9002-93-1) for 10 min, and the cells were washed with cold PBS three times. The fixed and permeabilized cells were blocked with PBST (1% BSA and 22.52 mg/mL Glycine) for 30 min. The anti-RAB7 AF647 antibody (Abcam, ab198337) and anti-Flag AF488 antibody (Proteintech, CL488-80010) were diluted with PBST (1% BSA and 22.52 mg/mL Glycine) and incubated with the cells for 2 h at room temperature. Nuclei were stained with 10 μg/mL Hoechst (Solarbio, C0031) for 30 min at room temperature and washed with PBS three times. The glass slides were sealed with Floureshield (Sigma-Aldrich, F6182), and co-localization of vaccines and RAB7 were observed using Nikon A1R HD25.

### Human DC isolation and maturation

PBMCs were isolated from healthy donors using Ficoll-Paque PLUS (Cytiva, 17144002). Briefly, 15 mL of Ficoll was gently layered with 30 mL of whole blood in a 50 mL centrifuge tube. The sample was centrifuged at 2000 rpm for 30 min at room temperature. The middle layer containing PBMCs was collected, washed twice with PBS, then once with RPMI-1640 medium. The suspended PBMCs were seeded into a 6-well plate, adjusted to a concentration of 5 × 10^6^ cells/mL and cultured for 2 h. The non-adherent cells in the supernatant were collected and cryopreserved in liquid nitrogen, while the adherent cells were washed twice with prewarmed PBS. Differentiation and maturation of DCs were induced using the ImmunoCult™ Dendritic Cell Culture Kit (STEMCELL, 10985). In brief, differentiation supplement was added to dendritic cell medium at a 1:100 ratio. Then, 2.5 mL of medium was added to each well and cultured for 3 days. The culture was replaced with fresh differentiation medium and cultured for an additional 2 days. On day 5, the maturation supplement was added to the medium at a 1:100 ratio to ensure that mature DCs could display LMP2A epitopes on the membrane. Simultaneously, vaccines were added to the medium and adjusted to a concentration of 1 μg/mL for an additional 2 days of culture.

### Detection of mature markers on DCs

Following the isolation and maturation of DCs, we assessed the expression level of the CD86 on the cell membrane as a measure of DC maturation. To specifically identify DCs, we employed the following methodology: initially, gating was conducted to exclude CD19^+^ or CD3^+^ cells to eliminate interference from B and T cells. Subsequently, HLA-DR-positive cells were gated to distinguish DCs. Following this, we compared the CD86 levels before and after the addition of the maturation supplement. The flow cytometry antibodies used are detailed above. In brief, adherent cells were collected and then stained with a mixture of antibodies, including anti-human CD19 APC, anti-human CD3 Pacific blue, anti-human HLA-DR PE and anti-human CD86 Cy5.5, for 20 min at room temperature. After twice washes with FACS buffer, the cells were suspended in 0.5 mL FACS buffer, and the expression of each marker was detected using ThermoFisher Attune NxT.

### Human DCs-T cell coculture

After maturation, antigen-presenting DCs were harvested and cocultured with autologous T cells at a ratio of 1:50. The cells were cultured in 5% FBS X-VIVO^TM^ 15 Serum-free Hematopoietic Cell Medium (Lonza, 02-060Q) supplemented with 10 IU/mL IL2 (GenScript, Z00368) for 9 days, with the medium replenished every 3 days. Following coculture, the T cells were divided into two groups. One portion of each sample was restimulated with the corresponding vaccine (10 μg/mL) for 24 h, with the addition of 2 μM Brefeldin A (Beyotime, S1536) for the final 5 h. The cells were then stained with anti-human CD3 APC and anti-human CD8 Pacific blue for 30 min at room temperature, washed twice with FACS buffer, fixed with IC Fixation buffer (Invitrogen, 00-8222) for 30 min in the dark, and permeabilized with Permeabilization buffer (Invitrogen, 00-8333) twice. Subsequently, the cells were stained with anti-human IFN-γ PE in Permeabilization buffer for 30 min, washed twice with Permeabilization buffer, and the expression level of IFN-γ was detected using flow cytometry.

The remaining cells were labeled with the CFSE Cell Division Tracker Kit (Biolegend, 423801) and restimulated with 10 μg/mL vaccine for an additional 6 days. The cells were cultured in 5% FBS X-VIVO^TM^ medium with 10 IU/mL IL2. T cell proliferation was evaluated by measuring the dilution of CFSE, and anti-human CD3 APC and anti-human CD8 Pacific blue were used to distinguish CD4^+^ and CD8^+^ T cells.

### TLR4 binding and activation assay

The recombinant vaccines were incubated with 293T/mTLR4 or 293T/hTLR4 cells at a concentration of 0.2 μM for 20 min. Detection was performed using anti-Flag AF647 antibody targeting Flag tag present in each vaccine, following the protocol outlined in the Flow cytometry section.

For activation assay, 0.5 × 10^6^ THP1 cells were incubated with 100 nM B-hEDA-T3-Fc or B-T3-Fc for 24 h, with PMA and ionomycin serving as positive controls. The culture supernatants were collected and analyzed for human TNF-α levels using the human TNF-α ELISA kit (Thermofisher, KHC3011).

### Animal studies

Animal experiments were conducted in accordance with ethical regulations for animal research. Six-week-old female C57BL/6J mice (Zhejiang Vital River Laboratories) were housed in a pathogen-free environment. In the preventive models, mice received subcutaneously immunization with 1 nmol of vaccines in 200 μL PBS, combined with of a 50% (v/v) mixture of Alhydrogel® adjuvant 2% (Invivogen, vac-alu-250) at weeks 0, 2, 4, with a 2-week interval between injections. Subsequently, MC38/LMP2A cells was inoculated at 1 × 10^6^ or TC1/LMP2A was injected at 2 × 10^6^ in the right flank 10 days after the last vaccination. In the therapeutic models, mice were immunized one day after MC38/LMP2A or TC1/LMP2A inoculation, receiving three administrations of vaccines with a 5-day interval. For the combination study, 50 μg/kg BMS-1 (Selleck, S7911) was injected intraperitoneally in 100 μL PBS after each vaccination. Tumor volume and body weight were monitored every three days until the mice either died or the tumor volume exceeded 2 cm^3^ (Guidelines for Endpoints in Animal Study Proposals 2019).

To validate the functional CD8 epitopes, mice (n = 3) were subcutaneously immunized with 50 μg of T3 peptide in 200 μL PBS, combined with a 50% (v/v) mixture of Alhydrogel® adjuvant 2% (Invivogen, vac-alu-250) at weeks 0, 2, 4, and 6. Three days after the final immunization, splenocytes were harvested for ELISpot analysis. Specifically, 0.4 × 10⁶ splenocytes from the indicated groups were stimulated with 10 μg/mL of Epi 1 to Epi 7 for 48 h, and spot-forming assays were conducted following the manufacturer's instructions.

### ELISpot

For the preventive models, spleens were collected from mice 10 days after the last vaccination. After homogenizing and lysing using RBC buffer (Biolegend, 420302), the splenocytes were washed twice with PBS, and resuspended in RPMI-1640 medium to adjust the concentration to 4 × 10^6^ cells/mL. The mouse IFN-γ ELISpot Kit (DAKEWE, DKW22-2000-096) plate was activated by adding 200 μL RPMI-1640 medium to each well and incubated for 10 min. Then, 100 μL of splenocytes was added to each well and stimulated with corresponding vaccines at 10 μg/mL for 48 h. After incubation, the culture was discarded, and 200 μL cold H_2_O was added for 10 min at 4 ℃ to lyse the cells. The plate was washed with the kit-provided Wash buffer six times. Biotin-anti-mouse IFN-γ antibody was then incubated for 1 h at 37 ℃. After discarding the antibody, the plate was washed six times with Wash buffer. Subsequently, HRP-streptavidin was incubated for 1 h at 37 ℃, and the plate was washed six times. AEC solution was added to form spots, and the results were analyzed. For the therapeutic models, spleens were collected 7 days after the last vaccination, and the procedures were the same as mentioned above.

### Intracellular cytokine staining

Spleen cells were collected as described earlier, and the concentration was adjusted to 4 × 10^6^ cells/mL. The cells were seeded into a 24-well plate at a volume of 1 mL per well and stimulated with corresponding vaccines at 10 μg/mL for 24 h. Brefeldin A was added at a concentration of 2 μM during the last 5 h of stimulation. Subsequently, the cells were collected, stained with anti-mouse CD3ε PE and anti-mouse CD8a FITC, washed with FACS buffer, fixed with IC Fixation buffer as previously mentioned, permeabilized, and then stained with anti-mouse IFN-γ APC. The expression level of IFN-γ was detected using flow cytometry.

### Cell-based ELISA

The 96-well plate was initially coated with 50 μg/mL poly-D lysine (Sigma-Aldrich, 27964-99-4) overnight at 4 ℃, and subsequently seeded with 0.03 × 10^6^ MC38/LMP2A or TC1/LMP2A cells in each well for 24 h culture. After washing twice with prewarmed PBS, the cells were fixed with 4% Paraformaldehyde Fix Solution (Biosharp, BL539A) for 15 min at room temperature. Following two PBS washes, excess paraformaldehyde was neutralized with PBS containing 0.1M Glycine for 30 min. To suppress innate peroxidase activity, the plate was treated with 3% H_2_O_2_ (PBS) for 5 min, followed by blocking with 2% BSA PBS for 1 h. The plate was then washed twice with PBS. Subsequently, serum collected 7 days after the last vaccination in either preventive or therapeutic models was serially diluted and added to the plate for a 1 h incubation. After incubation, the plate was washed three times with PBS and incubated with HRP-goat anti-mouse IgG (Trans, HS201-01) for 1 h. Following another wash cycle with PBS three times, TMB Substrate (Elabscience, E-IR-R201) was added to initiate the reaction. The reaction was terminated with 2 M H_2_SO_4_, and the absorbance was measured at OD_450nm_.

### Immunohistochemistry study

The immunohistochemistry study aimed to evaluate the infiltration of mouse CD3^+^ T cells. Briefly, tumor tissues were embedded in paraffin, sliced into 4 μm sections, and mounted on glass slides. These sections underwent staining with anti-CD3 epsilon antibody (abcam, ab5690) overnight at 4 ℃. Subsequently, they were treated with HRP-goat anti-rabbit antibody (Trans, HS101) for 40 min at 37 ℃, followed by the addition of 3,3'-diaminobenzidine (YEASEN, 36201ES03). Finally, the tissue sections were analyzed based on the staining intensity to assess the presence and distribution of CD3^+^ T cells within the tumor tissues.

### Statistical analysis

The data were presented as mean ± SD from a minimum of 3 independent experiments with biological replicates, and the specific sample size (n) was indicated in the figure legend. Statistical analysis was performed using GraphPad prism 8 with two-way ANOVA Tukey's multiple comparisons test, and significance was determined when the p-value was below 0.05 (* p < 0.05, ** p < 0.01, *** p < 0.001, **** p < 0.0001).

## Supplementary Material

Supplementary figures and tables.

## Abbreviations

CTL: cytotoxic T lymphocyte
CFSE: carboxyfluorescein succinimidyl ester
DCs: dendritic cells
EBV: Epstein‒Barr virus
EERs: epitope enrichment regions
EDA: extra domain A of fibronectin
hEDA: human fibronectin EDA
HTL: helper T lymphocyte
IgG1: immunoglobulin G1
IEDB: Immune Epitope Database
MHC: major histocompatibility complex
NPC: nasopharyngeal carcinoma
PRRs: pattern recognition receptors
TLRs: Toll-like receptors
