# Supplementary material for: Designing multifunctional recombinant vaccines: an engineering strategy based on innovative epitope prediction-guided splicing
Source: Theranostics. 2025 Mar 3;15(9):3924–42. doi: 10.7150/thno.103755 (PMC11980677; doi:10.7150/thno.103755)
Supplement: Supplementary file 1 — Supplementary figures and tables. [file thnov15p3924s1.pdf]

Designing multifunctional recombinant vaccines: an engineering strategy based on innovative epitope prediction-guided splicing

Zhidong Wang[1], Xiaolin Yang[1], Xiaoyi Wei[1], Licai Shi[1], Xuechun Wang[1], Zhenjian Zhuo[1], Hailong Su[2], Wengao Wu[3], Yu J. Cao[1,4,*]

1. State Key Laboratory of Chemical Oncogenomics, Shenzhen Key Laboratory of Chemical Genomics, Peking University Shenzhen Graduate School, Shenzhen, Guangdong, 518055, China.
2. School of Laboratory Medicine and Biotechnology, Southern Medical University, Guangzhou, China.
3. Department of Cardiovascular Surgery, Yueyang Central Hospital, Yueyang, Hunan, 414000, China.
4. Institute of Chemical Biology, Shenzhen Bay Laboratory, Shenzhen, 518132, China.

* To whom correspondence should be addressed: joshuacao@pku.edu.cn

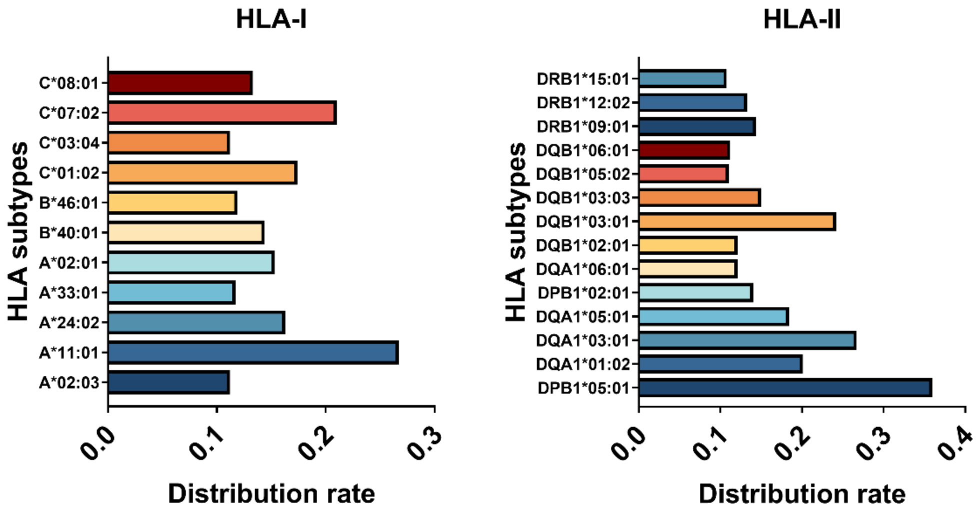

Figure S1. Prevalent HLA subtypes in Southeast Asia. The distribution rates of HLA subtypes with rates exceeding 10% in Southeast Asia are presented in two separate sections: HLA-I and HLA-II.

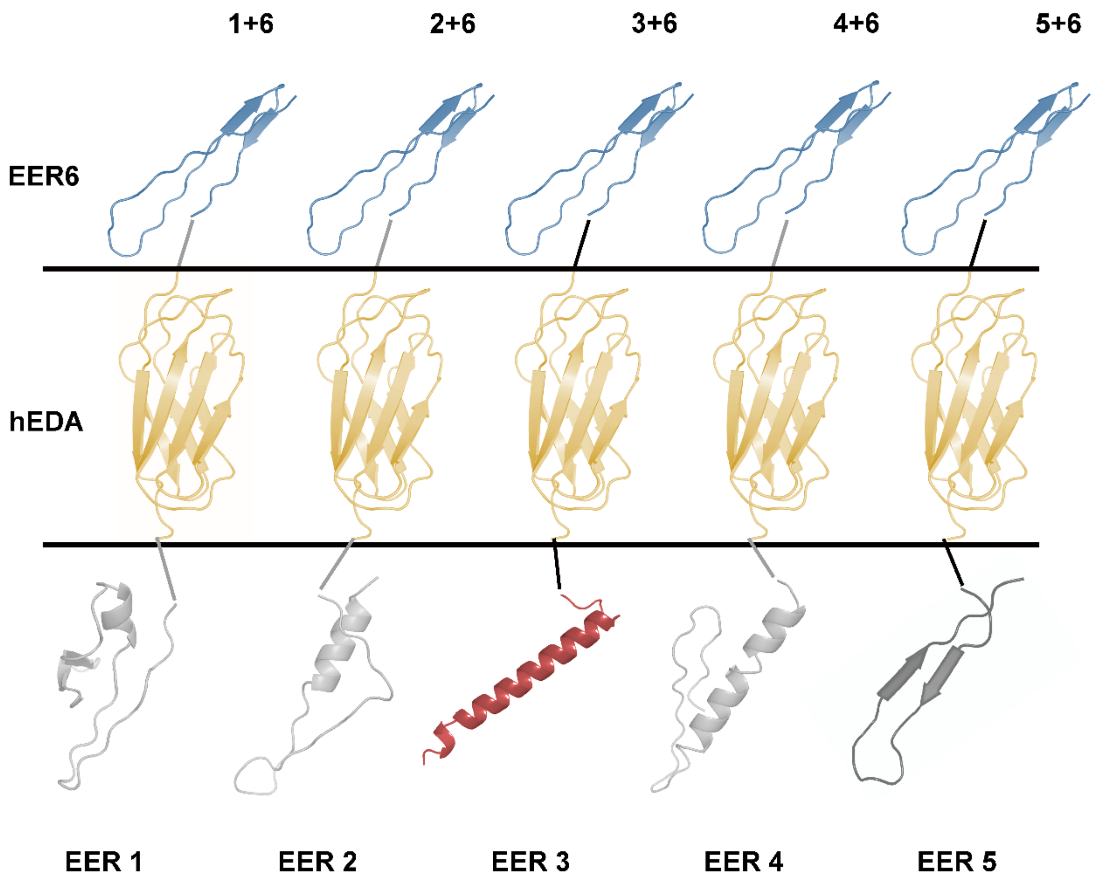

Figure S2. Predicted structures of five recombinant vaccines. The diagram illustrates the predicted structures of five recombinant vaccines (excluding the Fc portion). EER 6 is located at the N-terminus to ensure full exposure of the B-cell epitope (199-209). The β-sheet-rich hEDA domain is positioned in the middle of the recombinant vaccines, with the EER 1-5 junction at the C-terminus. Rigid linkers, composed of EAAAK sequences, were used to connect these three segments. The structures were predicted using Protein Homology/analog Recognition Engine V 2.0 and visualized with PyMOL.

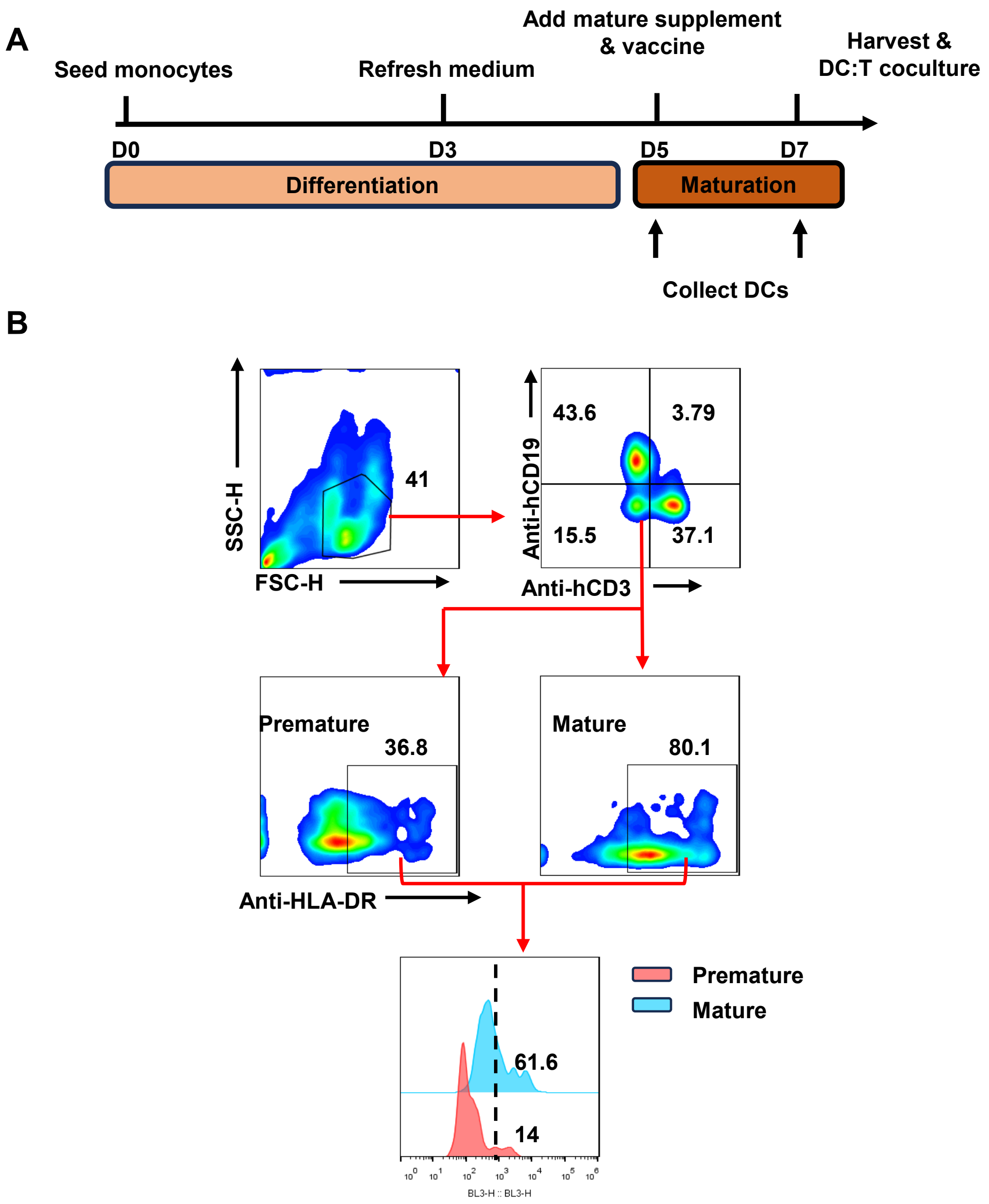

Figure S3. Detection of maturation markers in dendritic cells (DCs). A) This diagram illustrates the process of DC isolation, differentiation, maturation, and co-culture. B) The strategy used to identify DCs from peripheral blood mononuclear cells (PBMCs); the expression level of CD86 in CD3$^-$CD19$^-$HLA-DR$^+$ cells was measured both before and after maturation. hCD19: human CD19; hCD3: human CD3; hCD86: human CD86.

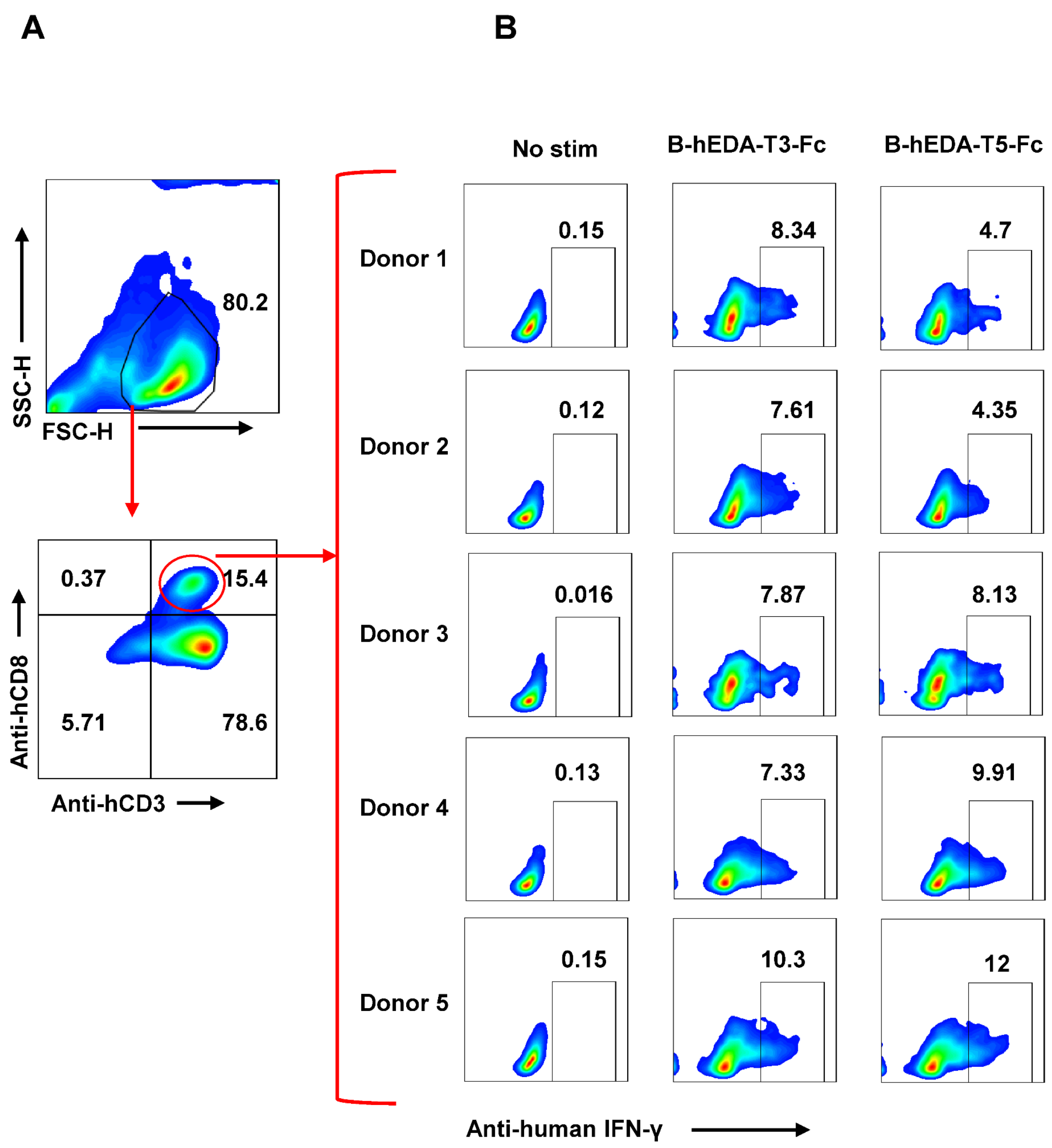

Figure S4. Detection of IFN-γ production in human CD8⁺ T Cells. A) The strategy for isolating CD3⁺ CD8⁺ T cells. B) Representative plots showing the detection of IFN-γ in CD8⁺ T cells from different donors after co-culture with vaccine-loaded dendritic cells (DCs) and restimulation with recombinant vaccines. hCD8: human CD8; hCD3: human CD3.

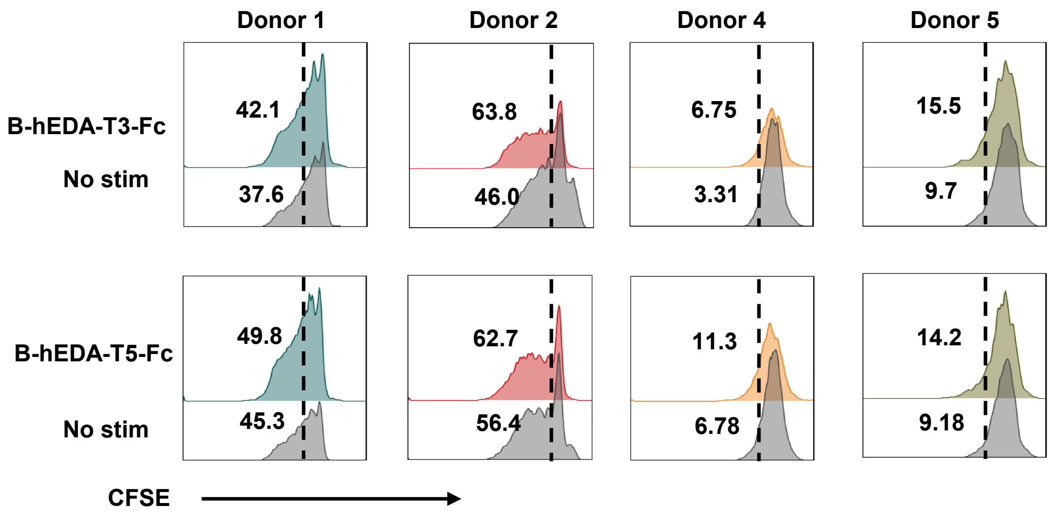

Figure S5. Detection of T cell proliferation. Representative results showing T cell division six days after stimulation. The "no stim" group refers to cells that were not restimulated with recombinant vaccine. Graphs were generated using FlowJo V10.

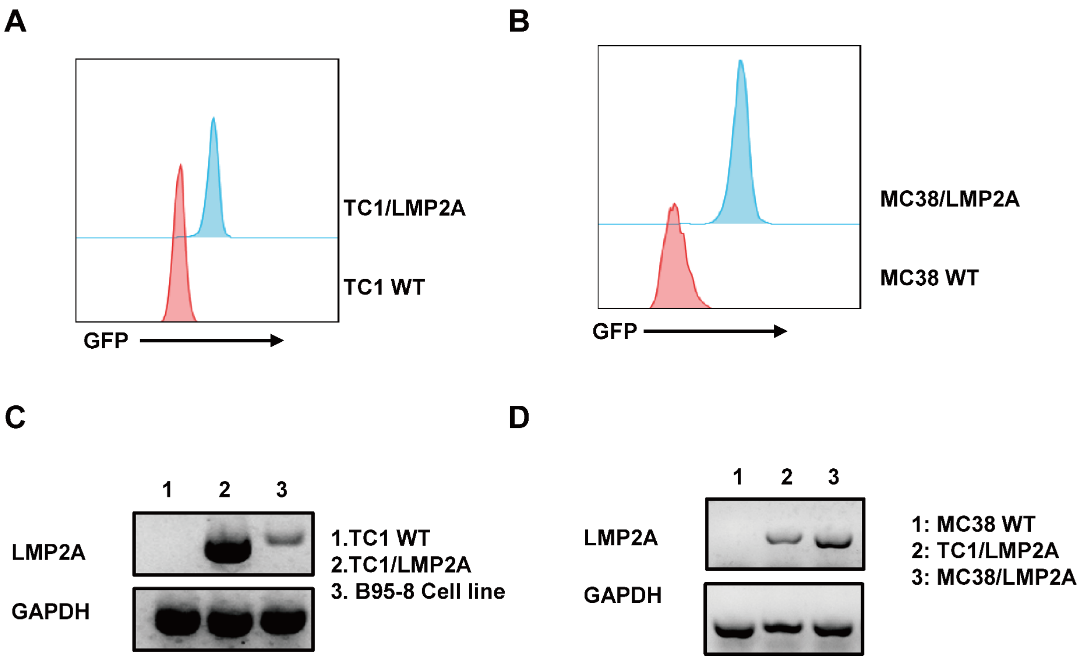

Figure S6. Validation of LMP2A-expressing mouse cell lines. The expression of GFP in LMP2A-transfected cell lines TC1/LMP2A (A) and MC38/LMP2A (B) was measured using flow cytometry. RT-PCR was used to detect the mRNA levels in transfected cell lines TC1/LMP2A (C) and MC38/LMP2A (D). The B95-8 cell line served as a positive control for TC1/LMP2A identification.

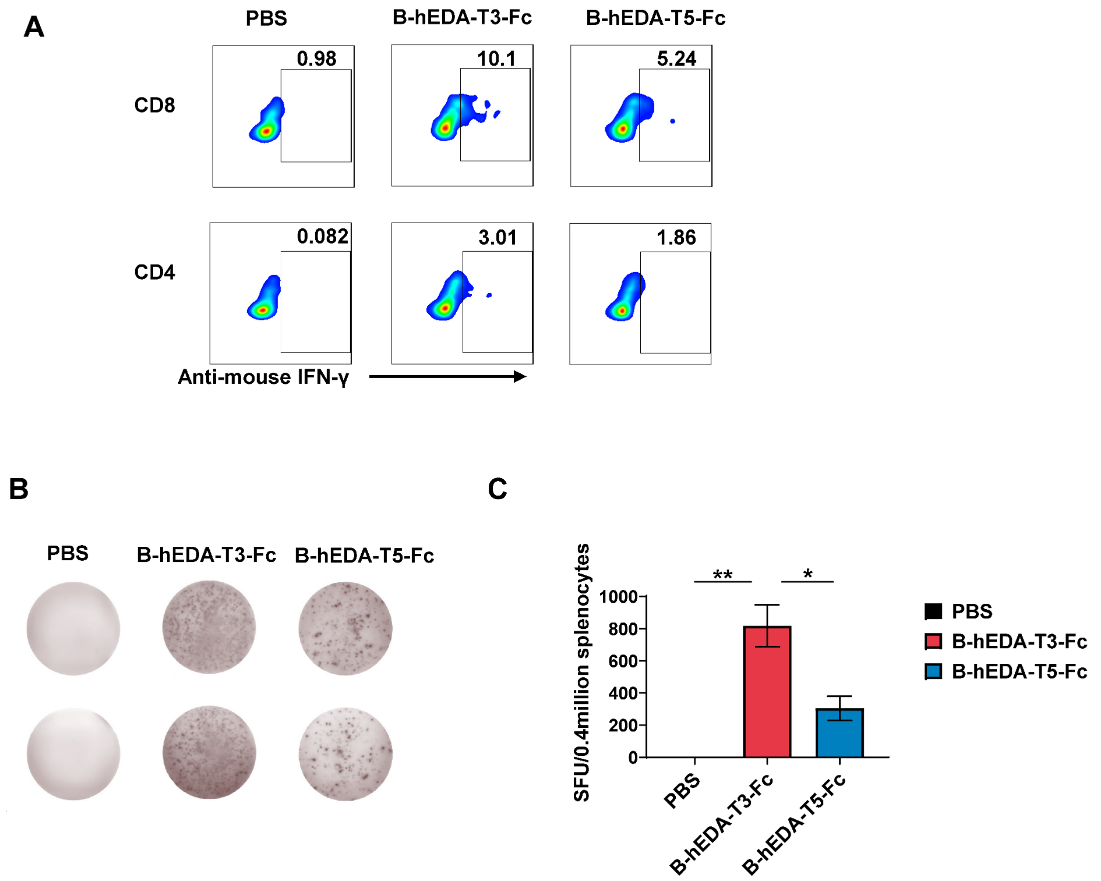

Figure S7. Detection of cellular immune response of B-hEDA-T3-Fc and B-hEDA-T5-Fc. C57BL/6J mice (n = 5) were immunized with B-hEDA-T3-Fc and B-hEDA-T5-Fc three times at 14-day intervals, with PBS as the control. Two mice from each group were randomly selected and sacrificed ten days after the final immunization. Splenocytes were then isolated, restimulated with the corresponding vaccines, and assayed for IFN-γ secretion using the ICS and ELISpot assay. A) Representative images of IFN-γ^{+} T cells detection in spleen. B) Representative ELISpot images after various stimulations. C) Quantification of spot-forming cells from panel A. Data were analyzed using ordinary one-way ANOVA with Tukey's multiple comparison test, with statistical significance set at p-values less than 0.05 (* $p < 0.05$, ** $p < 0.01$, *** $p < 0.001$, **** $p < 0.0001$).

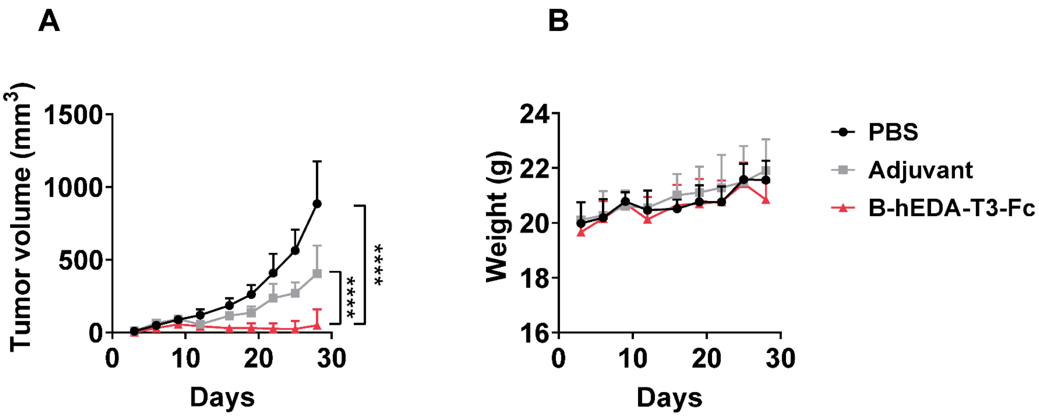

Figure S8. Inhibition of MC38/LMP2A tumor growth after B-hEDA-T3-Fc immunization. In the MC38/LMP2A preventive assay (n = 5), mice were immunized subcutaneously with 1 nmol of B-hEDA-T3-Fc three times, with two-week intervals. Ten days after the final immunization, 1×10^6 MC38/LMP2A cells were inoculated into the right flank. A) Tumor volume of MC38/LMP2A and B) body weight of the mice was monitored throughout the experiment. Data were analyzed using ordinary two-way ANOVA with Tukey's multiple comparison test, with statistical significance set at p-values less than 0.05 (* $p < 0.05$, ** $p < 0.01$, *** $p < 0.001$, **** $p < 0.0001$).

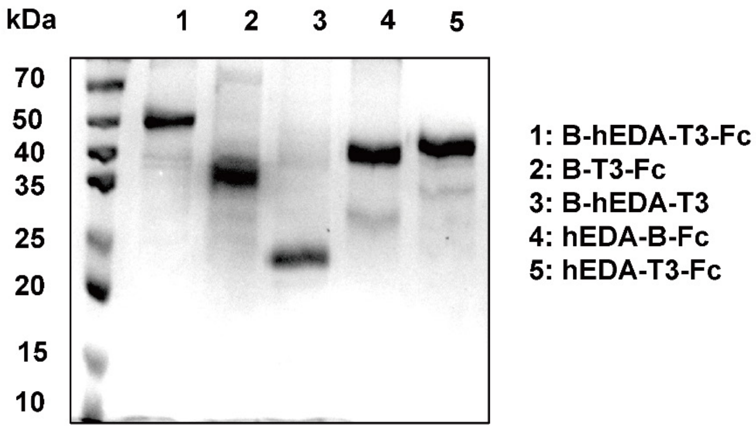

Figure S9. SDS-PAGE analysis showing protein profiles of B-hEDA-T3-Fc, B-T3-Fc, B-hEDA-T3, hEDA-B-Fc, and hEDA-T3-Fc.

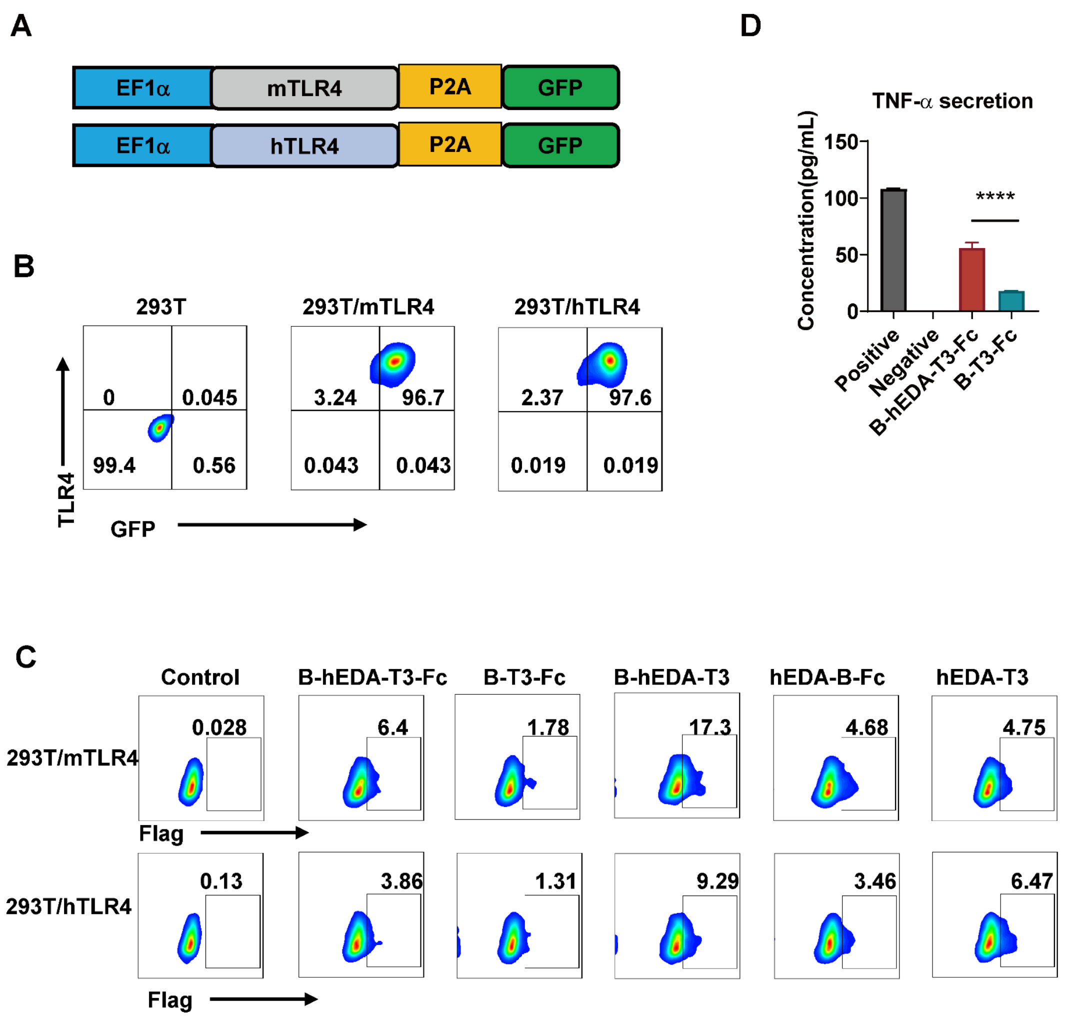

Figure S10. Generation of TLR4-expressing 293T cell lines and functional validation of recombinant vaccines. A) Expression cassette of genes associated with TLR4 expression. B) Flow cytometry analysis of mTLR4 and hTLR4 expression on engineered 293T cells. C) Representative images showing vaccine binding detection on TLR4-expressing cells. D) TNF-α secretion results in THP1 cells following stimulation. Data were analyzed using ordinary one-way ANOVA with Tukey's multiple comparison test, with statistical significance set at p-values less than 0.05 (* $p < 0.05$, ** $p < 0.01$, *** $p < 0.001$, **** $p < 0.0001$).

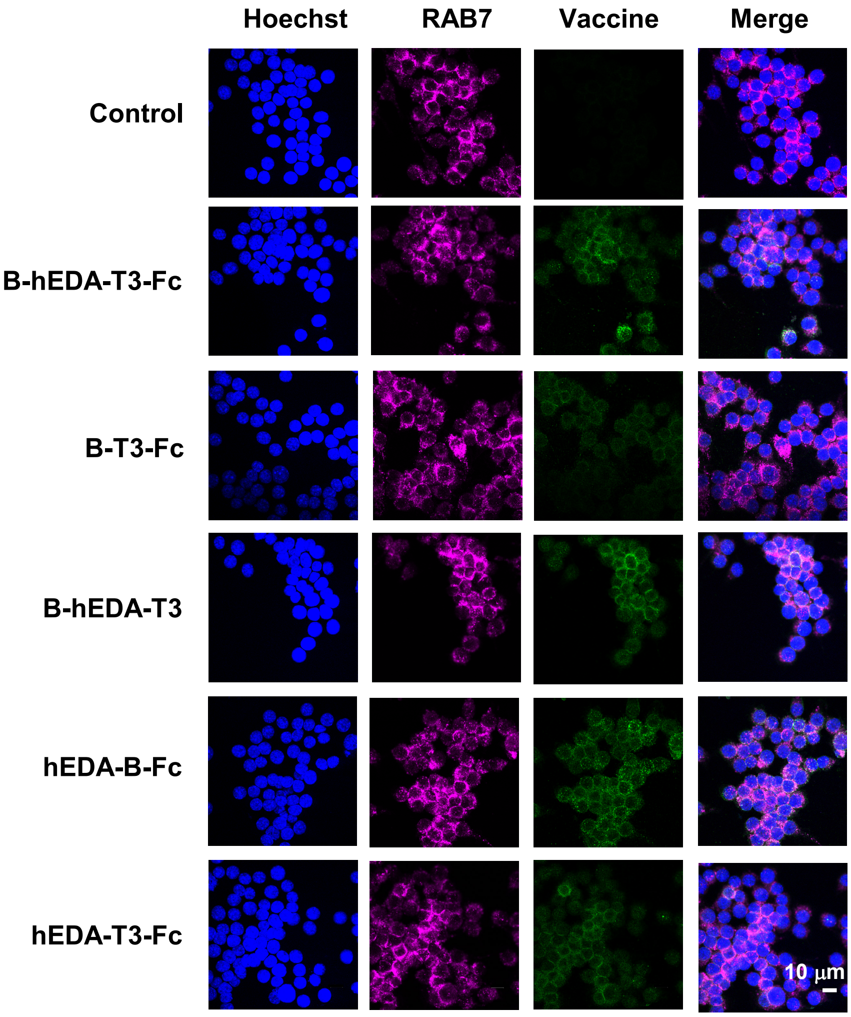

Figure S11. Comparison of antigen uptake by RAW264.7 cells between B-hEDA-T3-Fc and split vaccines using immunofluorescence staining. Representative images show the co-localization of recombinant vaccines. A Flag tag was added to the C-terminal of hEDA-B-Fc, hEDA-T3-Fc, and B-hEDA-T3, while B-T3-Fc had a Flag tag at the N-terminal. 0.5×10^6 RAW264.7 cells were cultured on slides for 24 h before the addition of 100 nM vaccines. After 1 h of culture, cells were fixed, permeabilized, and stained. (Magenta: AF647-anti-RAB7; Blue: Hoechst; Green: AF488-anti-Flag).

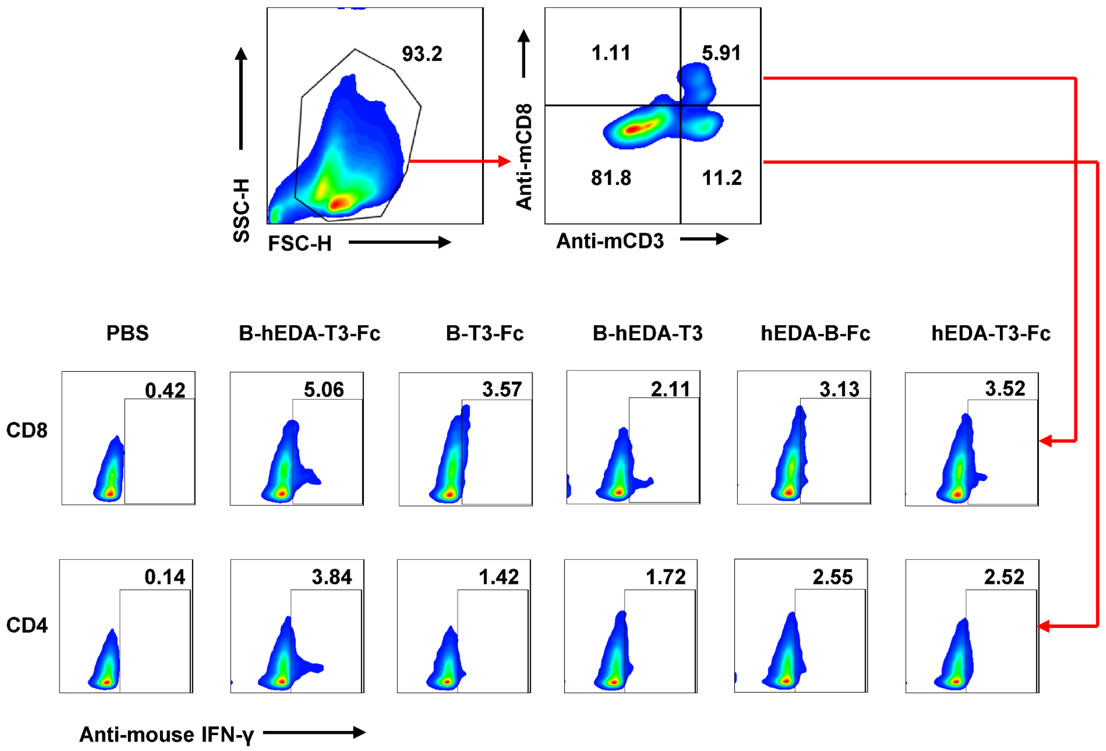

Figure S12. Comparison of IFN-γ$^+$ T cells in splenocytes after immunization with B-hEDA-T3-Fc and split vaccines. Splenocytes were collected seven days after the third vaccination in the MC38/LMP2A therapeutic assay, and the detection of IFN-γ$^+$ was carried out in both CD4$^+$ and CD8$^+$ T cells. mCD8: mouse CD8; mCD3: mouse CD3.

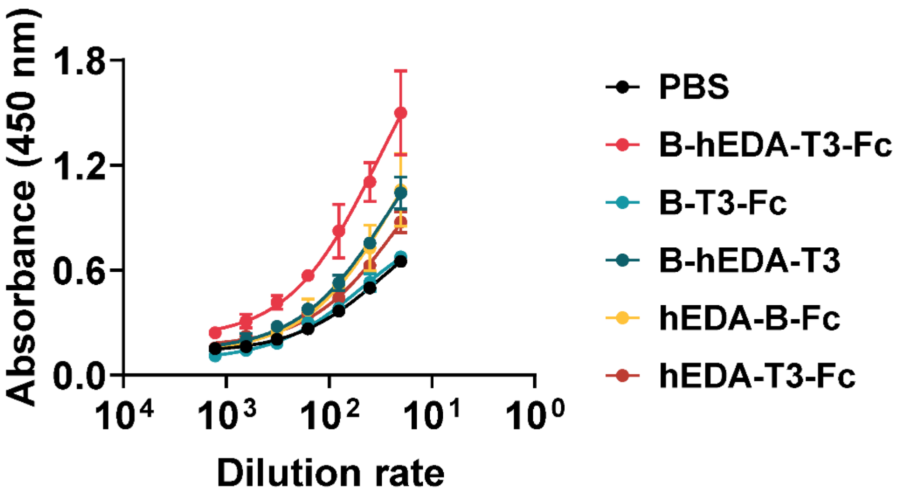

Figure S13. Serum antibody titration of B-hEDA-T3-Fc and split vaccines. Female C57BL/6J mice (n = 3) were immunized three times with a 2-week interval. Seven days after the last immunization, serum samples were collected and analyzed using MC38/LMP2A cell-based ELISA. The dilution gradient ranged from 1 : 20 to 1 : 1280.

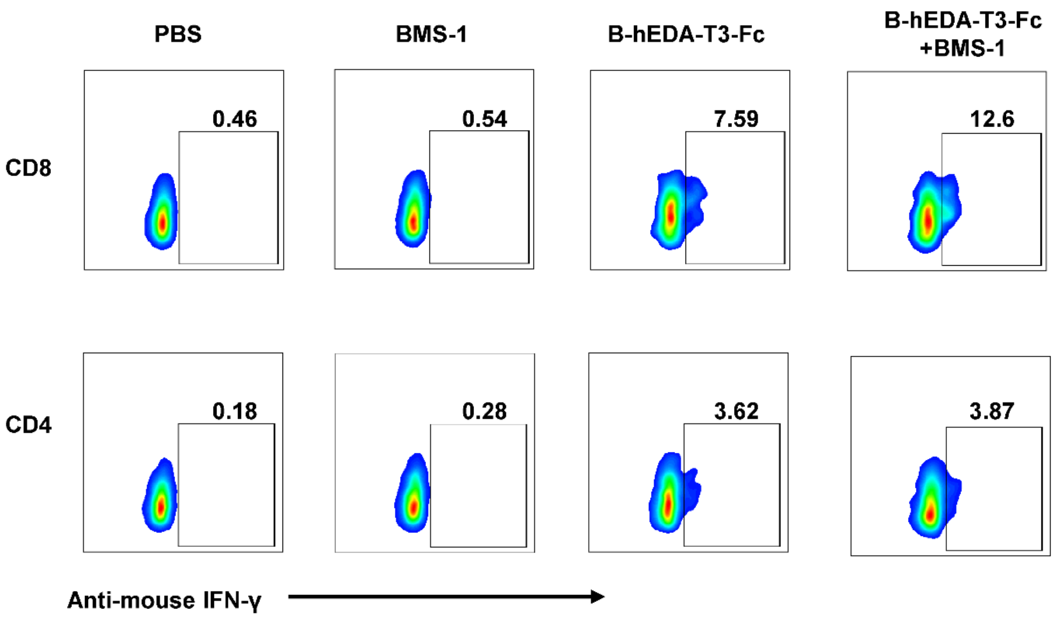

Figure S14. Representative plots for IFN-γ⁺ splenocytes detection in vaccine and BMS-1 synergy experiment. Splenocytes were collected seven days after the last immunization in the MC38/LMP2A therapeutic model. IFN-γ^+ detection was conducted in both CD4$^+$ and CD8$^+$ T cells using flow cytometry.

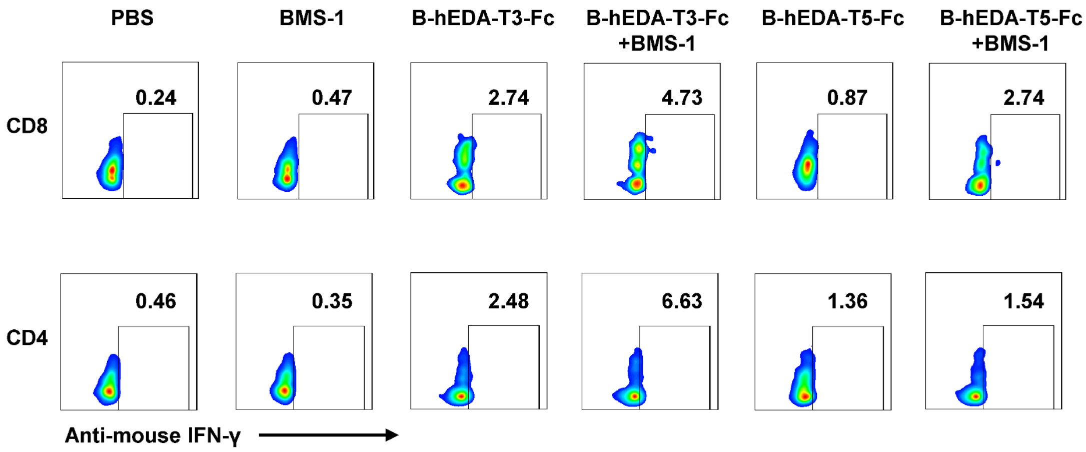

Figure S15. Representative plots for IFN-γ^{+} splenocytes detection in the TC1/LMP2A therapeutic model. Splenocytes were collected seven days after the last immunization in the TC1/LMP2A therapeutic model. The cells were then separated into $CD8^{+}$ and $CD4^{+}$ T cell populations, and IFN-γ^{+} detection was conducted using flow cytometry.

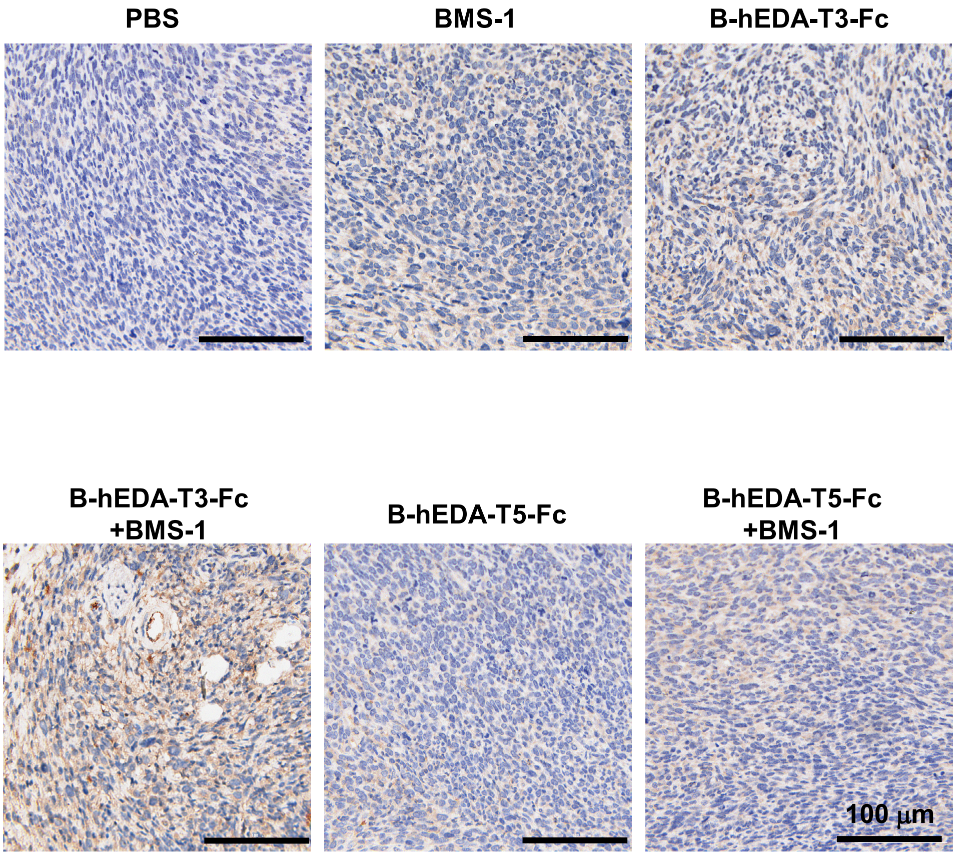

Figure S16. $CD3^+$ T cell infiltration in TC1/LMP2A tumor tissue. TC1/LMP2A tumors were harvested on day 28. Immunohistochemical staining was used to analyze the distribution of $CD3^+$ T cells within the tumor tissue. The scale bar represents 100 μm. Representative plots are shown.

Table S1 Summary of predicted CTL epitopes

Position	Sequence	Length	Antigenicity prediction score	Toxicity	Allergenicity
112-120	YEEAGRGSM	9	-0.1523 (Probable NON-ANTIGEN)	Non-Toxin	PROBABLE ALLERGEN
119-127	SMNPVCLPV	9	1.5822 (Probable ANTIGEN)	Non-Toxin	PROBABLE NON-ALLERGEN
126-136	PVIVAPYLFWL	11	0.4615 (Probable ANTIGEN)	Non-Toxin	PROBABLE ALLERGEN
132-140	YLFWLAAIA	9	0.6544 (Probable ANTIGEN)	Non-Toxin	PROBABLE NON-ALLERGEN
144-152	FTASVSTVV	9	0.1138 (Probable NON-ANTIGEN)	Non-Toxin	PROBABLE ALLERGEN
156-164	GLALSLLLL	9	0.6946 (Probable ANTIGEN)	Non-Toxin	PROBABLE NON-ALLERGEN
163-171	LLAAVASSY	9	0.4839 (Probable ANTIGEN)	Non-Toxin	PROBABLE NON-ALLERGEN
166-178	AVASSYAAAQRKL	13	0.4955 (Probable ANTIGEN)	Non-Toxin	PROBABLE NON-ALLERGEN
177-185	KLLTPVTVL	9	-0.1523 (Probable NON-ANTIGEN)	Non-Toxin	PROBABLE ALLERGEN
183-191	TVLTAVVTF	9	0.2199 (Probable NON-ANTIGEN)	Non-Toxin	PROBABLE NON-ALLERGEN
190-198	TFFAICLTW	9	1.6665 (Probable ANTIGEN)	Non-Toxin	PROBABLE ALLERGEN
199-209	RIEDPPFNSLL	11	0.7161 (Probable ANTIGEN)	Non-Toxin	PROBABLE NON-ALLERGEN
207-216	SLLFALLAAA	10	0.5835 (Probable ANTIGEN)	Non-Toxin	PROBABLE NON-ALLERGEN
211-223	ALLAAAGGLQGIY	13	0.3651 (Probable NON-ANTIGEN)	Non-Toxin	PROBABLE NON-ALLERGEN
218-226	GLQGIYVLV	9	0.6584 (Probable ANTIGEN)	Non-Toxin	PROBABLE NON-ALLERGEN
254-265	VLVLIVDAVLQL	12	0.2855 (Probable NON-ANTIGEN)	Non-Toxin	PROBABLE NON-ALLERGEN
261-275	AVLQLSPLLGAVTVV	15	0.9421 (Probable ANTIGEN)	Non-Toxin	PROBABLE ALLERGEN
293-304	GLGTLGAALLTL	12	0.8335 (Probable ANTIGEN)	Non-Toxin	PROBABLE NON-ALLERGEN
300-311	ALLTLAAALALL	12	0.5058 (Probable ANTIGEN)	Non-Toxin	PROBABLE NON-ALLERGEN
307-315	ALALLASLI	9	0.2894 (Probable NON-ANTIGEN)	Non-Toxin	PROBABLE NON-ALLERGEN
310-321	LLASLILGTLNL	12	0.6677 (Probable ANTIGEN)	Non-Toxin	PROBABLE NON-ALLERGEN
318-326	TLNLTTMFL	9	0.4943 (Probable ANTIGEN)	Non-Toxin	PROBABLE NON-ALLERGEN
329-337	LLWTLVVLL	9	0.6089 (Probable ANTIGEN)	Non-Toxin	PROBABLE NON-ALLERGEN
354-365	RLFLYALALLLL	12	0.0267 (Probable NON-ANTIGEN)	Non-Toxin	PROBABLE NON-ALLERGEN

368-376	ALIAGGSIL	9	0.1875 (Probable NON-ANTIGEN)	Non-Toxin	PROBABLE NON-ALLERGEN
373-383	GSILQTNFKSL	11	0.8458 (Probable ANTIGEN)	Non-Toxin	PROBABLE NON-ALLERGEN
399-407	IVAGILFIL	9	0.2860 (Probable NON-ANTIGEN)	Non-Toxin	PROBABLE NON-ALLERGEN
419-427	TYGPVFMCL	9	0.6292 (Probable ANTIGEN)	Toxin	PROBABLE NON-ALLERGEN
426-434	CLGGLLTMV	9	0.2345 (Probable NON-ANTIGEN)	Non-Toxin	PROBABLE ALLERGEN
429-442	GLLTMVAGAVWLTV	14	0.2024 (Probable NON-ANTIGEN)	Non-Toxin	PROBABLE ALLERGEN
442-450	VMSNTLLSA	9	0.0294 (Probable NON-ANTIGEN)	Non-Toxin	PROBABLE NON-ALLERGEN
447-455	LLSAWILTA	9	-0.0037 (Probable NON-ANTIGEN)	Non-Toxin	PROBABLE NON-ALLERGEN
459-469	IFLIGFALFGV	11	0.6914 (Probable ANTIGEN)	Non-Toxin	PROBABLE NON-ALLERGEN

Table S2 Summary of predicted HTL epitopes

Allele	Position	Sequence	Length	Antigenicity prediction score	Toxicity	Allergenicity
HLA-DQA1*05:01/DQB1*03:01	3-21	SLEMVPMGAGPPSPGGDPD	19	0.8489 (Probable ANTIGEN)	Non-Toxin	PROBABLE ALLERGEN
HLA-DQA1*05:01/DQB1*03:01, HLA-DRB1*09:01	127-159	VIVAPYLFWLAAIAASCFTASVSTVVTATGLAL	33	0.5902 (Probable ANTIGEN)	Non-Toxin	PROBABLE NON-ALLERGEN
HLA-DQA1*05:01/DQB1*03:01	148-163	VSTVVTATGLALSLLL	16	0.6814 (Probable ANTIGEN)	Non-Toxin	PROBABLE NON-ALLERGEN
HLA-DQA1*05:01/DQB1*03:01	157-179	LALSLLLLAAVASSYAAAQRKLL	23	0.5027 (Probable ANTIGEN)	Non-Toxin	PROBABLE NON-ALLERGEN
HLA-DPA1*01:03/DPB1*02:01, HLA-DQA1*05:01/DQB1*03:01	199-227	RIEDPPFNSLLFALLAAAGGLQGIYVLVM	29	0.5416 (Probable ANTIGEN)	Non-Toxin	PROBABLE ALLERGEN
HLA-DRB1*15:01	223-241	YVLVMLVLLILAYRRRWRR	19	0.5247 (Probable ANTIGEN)	Non-Toxin	PROBABLE NON-ALLERGEN
HLA-DQA1*05:01/DQB1*03:01	262-280	VLQLSPLLGAVTVVSMTLL	19	0.9386 (Probable ANTIGEN)	Non-Toxin	PROBABLE ALLERGEN
HLA-DRB1*15:01, HLA-DPA1*01:03/DPB1*02:01	275-291	VSMTLLLLAFVLWLSSP	17	0.3472 (Probable NON-ANTIGEN)	Non-Toxin	PROBABLE NON-ALLERGEN
HLA-DQA1*05:01/DQB1*03:01, HLA-DRB1*09:01, HLA-DRB1*15:01	288-320	LSSPGGLGTLGAALLTLAAALALLASLILGTLN	33	0.6164 (Probable ANTIGEN)	Non-Toxin	PROBABLE NON-ALLERGEN
HLA-DRB1*15:01	321-339	LTTMFLLMLLWTLVVLLIC	19	0.3479 (Probable NON-ANTIGEN)	Non-Toxin	PROBABLE NON-ALLERGEN
HLA-DPA1*01:03/DPB1*02:01, HLA-DRB1*15:01, HLA-DQA1*05:01/DQB1*03:01	345-383	CPLSKILLARLFLYALALLLLASALIAGGSILQTNFKSL	39	0.1645 (Probable NON-ANTIGEN)	Non-Toxin	PROBABLE NON-ALLERGEN

HLA-DRB1*09:01	374-392	SILQTNFKSLSSTEFIPNL	19	0.7309 (Probable ANTIGEN)	Non-Toxin	PROBABLE ALLERGEN
HLA-DPA1*01:03/DPB1*02:01	382-400	SLSSTEFIPNLFCMLLLIV	19	0.7816 (Probable ANTIGEN)	Non-Toxin	PROBABLE NON-ALLERGEN
HLA-DRB1*15:01	391-414	NLFCMLLLIVAGILFILAILTEWG	24	0.5499 (Probable ANTIGEN)	Non-Toxin	PROBABLE NON-ALLERGEN
HLA-DQA1*05:01/DQB1*03:01	427-444	LGGLLTMVAGAVWLTVMS	18	0.1938 (Probable NON-ANTIGEN)	Non-Toxin	PROBABLE NON-ALLERGEN
HLA-DRB1*15:01	452-470	ILTAGFLIFLIGFALFGVI	19	0.5036 (Probable ANTIGEN)	Non-Toxin	PROBABLE NON-ALLERGEN

Table S3 Summary of predicted linear B cell epitopes

No.	Start	End	Peptide	Length	Located in loops
1	9	81	MGAGPPSPGGDPDGYDGGNNSQYPSASGSSGNTPTPPNDEERESNEEPPPP YEDPYWGNGDRHSDYQPLGTQD	73	No
2	90	120	HDGNDGLPPPPYSPRDDSSQHIYEEAGRGSM	31	No
3	171	173	YAA	3	No
4	201	205	EDPPF	5	Yes (loop 2)
5	291	296	PGGLGT	6	No
6	413	420	WGSGNRTY	8	No

Table S4. Summary of predicted epitope enrichment regions (EERs) of LMP2A.

EER	Position	Sequence	HLA isotypes	Length	Epitope density per amino acid length
EER 1	119-163	SMNPVCLPVIVAPYLFW LAAIAASCFTASVSTVV TATGLALSLLL	119-127 A02<br>126-136 A24<br>132-140 A02<br>144-152 A02<br>127-159 HLA-DQA1*05:01/DQB1*03:01, HLA-DRB1*09:01<br>148-163 HLA-DQA1*05:01/DQB1*03:01	45aa	6/45=0.133
EER 2	254-291	VLVLIVDAVLQLSPLLG AVTVVSMTLLLLAFVL WLSSP	254-265 A02<br>261-275 A02<br>262-280 HLA-DQA1*05:01/DQB1*03:01<br>275-291 HLA-DRB1*15:01, HLA-DPA1*01:03/DPB1*02:01	38aa	4/38=0.105
EER 3	288-326	LSSPGGLGTLGAALLTL AAALALLASLILGTLNL TTMFL	293-304 A02<br>300-311 A02<br>307-315 A02<br>310-321 A02<br>318-326 A02<br>288-320 HLA-DQA1*05:01/DQB1*03:01, HLA-DRB1*09:01	39aa	6/39=0.1538
EER 4	345-392	CPLSKILLARLFLYALAL LLLASALIAGGSILQTNF KSLSSTEFIPNL	345-383 HLA-DPA1*01:03/DPB1*02:01, HLA-DRB1*15:01<br>354-365 A02<br>368-376 A02<br>373-383 A11<br>374-392 HLA-DRB1*09:01	48aa	5/48=0.104

EER 5	419-444	TYGPVFMCLGGLLTMV AGAVWLTVMS	419-427 A24<br>426-434 A02<br>429-442 A02<br>427-444 HLA-DQA1*05:01/DQB1*03:01	26aa	4/26=0.1538
EER 6	190-227	TFFAICLTWRIEDPPFNS LLFALLAAAGGLQGIYV LVM	190-198 A24<br>199-209 B40<br>B cell epitope<br>211-223 A02 B15<br>207-216 A02<br>218-226 A02<br>199-227 HLA-DPA1*01:03/DPB1*02:01, HLA-DQA1*05:01/DQB1*03:01	38aa	7/38=0.184

This table summarizes the positions, sequences, HLA isotypes contained within the EERs, and the HLA coverage for all predicted EERs.

Table S5. Summary of HLA isotypes for five healthy donors.

Sample ID	HLA-A/B	HLA-DQA1	HLA-DQB1	HLA-DRB1
Donor 1	A02:01,11:01	03:01,03:03	03:02,04:01	04:05,04:06
Donor 2	A02:03,33:03	01:01,05:01	05:01,02:01	03:01,15:02
Donor 3	B40:01,39:01	01:02,01:04	05:03,05:03	14:05,15:01
Donor 4	A11:01,24:02	01:02,03:01	05:02,03:02	04:03,16:02
Donor 5	A24:02,29:01	05:05,05:05	03:01,03:01	11:01,11:01

Table S6. Summary of predicted results for B, T3, and T5 with C57BL/6J MHC: H-2Db.

EER	Allele	Start	End	Length	Peptide	Score
EER3 (T3)	H-2-Db	29	37	9	LGTLNLTTM	0.716486
	H-2-Db	19	27	9	AALALLASL	0.569715
	H-2-Db	25	37	13	ASLILGTLNLTTM	0.485801
	H-2-Db	2	10	9	SSPGGLGTL	0.401646
	H-2-Db	26	37	12	SLILGTLNLTTM	0.317338
	H-2-Db	28	37	10	ILGTLNLTTM	0.276769
	H-2-Db	27	37	11	LILGTLNLTTM	0.192672
	H-2-Db	30	37	8	GTLNLTTM	0.165304
	H-2-Db	26	34	9	SLILGTLNL	0.101483
	H-2-Db	29	39	11	LGTLNLTTMFL	0.094921
EER5 (T5)	H-2-Db	14	22	9	TMVAGAVWL	0.100492
	H-2-Db	17	25	9	AGAVWLTVM	0.082066
	H-2-Db	6	15	10	FMCLGGLLTM	0.04988
	H-2-Db	7	15	9	MCLGGLLTM	0.042153
	H-2-Db	16	24	9	VAGAVWLTV	0.027504
	H-2-Db	2	9	8	YGPVFMCL	0.016949
	H-2-Db	2	15	14	YGPVFMCLGGLLTM	0.016367
	H-2-Db	2	12	11	YGPVFMCLGGL	0.009475
	H-2-Db	16	25	10	VAGAVWLTVM	0.008367
	H-2-Db	2	13	12	YGPVFMCLGGLL	0.007389
EER6 (B)	H-2-Db	28	36	9	GGLQGIYVL	0.185527
	H-2-Db	26	36	11	AAGGLQGIYVL	0.184472
	H-2-Db	3	11	9	FAICLTWRI	0.126086
	H-2-Db	25	36	12	AAAGGLQGIYVL	0.046525
	H-2-Db	27	36	10	AGGLQGIYVL	0.04399
	H-2-Db	16	24	9	FNSLLFALL	0.031904
	H-2-Db	11	19	9	IEDPPFNSL	0.030418
	H-2-Db	27	35	9	AGGLQGIYV	0.02981
	H-2-Db	11	21	11	IEDPPFNSLLF	0.029
	H-2-Db	25	33	9	AAAGGLQGI	0.026172

Predictions were made for B, T3, and T5 with H-2Db, listing the top 10 high-scoring epitopes. The table includes the peptide positions, sequences, and scores.

Table S7. Details of antibodies used in this study.

Reagent	Source	Identifier
Anti-human CD3 APC	Biolegend	300312
Anti-human CD19 APC	Biolegnd	302212
Anti-human CD3 Pacific blue	Biolegend	300330
Anti-human CD8 Pacific blue	Biolegend	344718
Anti-human IFN-γ PE	Biolegend	506507
Anti-human HLA-DR PE	Biolegend	307606
Anti-mouse CD3ε PE	Biolegend	100308
Anti-mouse CD8a FITC	Biolegend	100706
Anti-mouse IFN-γ APC	Invitrogen	17-7311-82
Anti-human CD86 Cy5.5	Biolegend	305420
Anti-RAB7 AF647	Abcam	ab198337
Anti-Flag AF488	Proteintech	CL488-80010
Anti-Flag AF647	Biolegend	362611
Anti-CD3 epsilon	Abcam	ab5690
HRP goat anti rabbit IgG (H+L)	TransGen Biotech	HS101
